# Molecular evolutionary and structural analysis of human *UCHL1* gene demonstrates the relevant role of intragenic epistasis in Parkinson’s disease and other neurological disorders

**DOI:** 10.1186/s12862-020-01684-7

**Published:** 2020-10-07

**Authors:** Muhammad Saqib Nawaz, Razia Asghar, Nashaiman Pervaiz, Shahid Ali, Irfan Hussain, Peiqi Xing, Yiming Bao, Amir Ali Abbasi

**Affiliations:** 1 National Center for Bioinformatics, Program of Comparative and Evolutionary Genomics, Faculty of Biological Sciences, Quaid-i-Azam University, Islamabad, 45320 Pakistan; 2 National Genomics Data Center & CAS Key Laboratory of Genome Sciences and Information, Beijing Institute of Genomics, Chinese Academy of Sciences and China National Center for Bioinformation, Beijing, 100101 China; 3 University of Chinese Academy of Sciences, Beijing, 100049 China

**Keywords:** UCHL1, Epistasis, Neurodegenerative diseases, Parkinson’s disease, SNCA

## Abstract

**Background:**

Parkinson’s disease (PD) is the second most common neurodegenerative disorder. PD associated human *UCHL1* (Ubiquitin C-terminal hydrolase L1) gene belongs to the family of deubiquitinases and is known to be highly expressed in neurons (1–2% in soluble form). Several functions of UCHL1 have been proposed including ubiquitin hydrolyze activity, ubiquitin ligase activity and stabilization of the mono-ubiquitin. Mutations in human *UCHL1* gene have been associated with PD and other neurodegenerative disorders. The present study aims to decipher the sequence evolutionary pattern and structural dynamics of UCHL1. Furthermore, structural and interactional analysis of UCHL1 was performed to help elucidate the pathogenesis of PD.

**Results:**

The phylogenetic tree topology suggests that the *UCHL1* gene had originated in early gnathostome evolutionary history. Evolutionary rate analysis of orthologous sequences reveals strong purifying selection on *UCHL1*. Comparative structural analysis of UCHL1 pinpoints an important protein segment spanning amino acid residues 32 to 39 within secretion site with crucial implications in evolution and PD pathogenesis through a well known phenomenon called intragenic epistasis. Identified critical protein segment appears to play an indispensable role in protein stability, proper protein conformation as well as harboring critical interaction sites.

**Conclusions:**

Conclusively, the critical protein segment of UCHL1 identified in the present study not only demonstrates the relevant role of intraprotein conformational epistasis in the pathophysiology of PD but also offers a novel therapeutic target for the disease.

## Background

Parkinson’s disease (PD) is the second most common neurodegenerative disorder after Alzheimer’s disease (AD) and is known to effect normal function of motor neurons [1]. PD prevalence is 1–2% of population above age 65 years and 4–5% above the age of 85 years [2]. There are two major pathological hallmarks of PD diagnosis, selective degeneration of dopaminergic neurons in substantia nigra (it’s a basal ganglia structure located in mid-brain that plays a key role in reward, addiction and movement) and the presence of an intracellular protein inclusion, lewy bodies (LBs) and lewy neuritis [2, 3]. PD-associated symptoms include motor features (rigidity, resting tremor, bradykinesia and postural instability) and non-motor features (olfactory dysfunction, autonomic dysfunction, cognitive impairment and psychiatric symptoms) [4]. Since 1997, 27 PD-associated genes have been identified with autosomal dominant (*UCHL1, GBA, GIGYF2, DNAJC13, LRRK2, TMEM230, GCH1, EIF4G1, SNCA, HTRA2, RIC3, ATXN2, VPS35, CHCHD2*), autosomal recessive (*PRKN, PTRHD1, DJ1, PLA2G6, SPG11, FBXO7, DNAJC6, SYNJ1, ATP13A2, VPS13C, PODXL, PINK1*) or an X-linked mode of transmission (*RAB39B*) [5]. *UCHL1* (Ubiquitin C-Terminal Hydrolase 1) is identified as a major causal gene involved in the early-onset of familial and sporadic PD and other neurodegenerative disorders like AD [6, 7] and Huntington’s disease [8]. As of now, five missense mutations in *UCHL1* has been associated with PD and other neurological disorders, i.e. E7A [7], S18Y [9], I93M [10], R178Q and A216D [11].

Ubiquitin C-terminal hydrolase L1 (UCHL1) is a 24.8 kDa, acidic protein (pI 5.3) [12], consisting of 223 amino acids and encoded by 9 exons with a transcript of 1172 bps in length, and located on human chromosome 4(4p14) [13]. UCHL1 (PGP 9.5 or PARK5) is the most abundant protein constituting 1–2% of the total brain soluble fraction, which is normally expressed exclusively in neurons and testis and is known to play a key role in ubiquitin turnover through its C-terminal hydrolase activity [3]. However, abnormal expression of UCHL1 is found in many primary lung tumors, lung tumor cell lines and colorectal cancers [14, 15]. UCHL1 functions as a de-ubiquitinating or hydrolase enzyme in the ubiquitin-proteasome pathway [11]. Furthermore, it also shows ubiquitin ligase activity for monoubiquitinated α-synuclein in a cell free system [16]. In addition, UCHL1 stabilizes the monoubiquitin or free ubiquitin and thus provides the availability of ubiquitin in various other cellular events independently of its enzymatic activity [17]. In the secondary structure comparison, circular dichroism analysis revealed that the mutant version of UCHL1 (I93M) has decreased level of alpha helix as compared to wild-type UCHL1, and therefore has a tendency to aggregate in neurons [18] and cause autosomal dominant form of PD [10]. Transgenic mouse analysis of mutant version of UCHL1 (I93M) exhibits the physiological phenotypes related to PD and degeneration of dopaminergic neurons within the age of 20 weeks [19]. Mutant version of UCHL1 (I93M) also exhibits increased insolubility, aberrant interactions with other proteins (HSP90 & HSC70) in mammalian cells and decreased interaction with monoubiquitin, suggesting that this mutant version (I93M) plays a causative role in familial PD [16]. Furthermore, in vitro analysis of recombinant UCHL1 (I93M) indicated the decline of 50% hydrolase activity or deubiquitinatinating activity as compared to the wild type [18, 20] which also contributes to the oxidative modification of UCHL1 [16, 19]. Intriguingly, subset of mutant versions of UCHL1 (S18Y) show lower risk of PD due to its reducing ligase activity that leads to reduced level of ubiquitinated alpha-synuclein [21]. However, some studies failed to identify these types of association between mutant version of UCHL1 (S18Y) and reduced risk of PD [22, 23]. At the molecular level, a missense mutant version of UCHL1 (E7A) exhibits extensive loss of ubiquitin binding ability thereby leading to completely abolished UCHL1 hydrolase activity [3, 7]. This loss of function can be rationalized at structural level because glutamic acid (at E7 position of wild type UCHL1) is located at ubiquitin binding interface to form electrostatic interactions with ubiquitin residues Arg42 and Arg72 [3]. A recent study analyzed the biochemical impact of the two PD causing UCHL1 missense mutations suggesting that mutant version of UCHL1 (R178Q) exhibits a 4-fold increased hydrolase activity as compared to the wild type. This increased enzymatic activity provides a protective effect on cognitive function. Another mutant version of UCHL1 (A216D) showed insolubility and consequently the complete loss of function [11].

Given the vital role of *UCHL1* in both familial and sporadic PD and other neurological disorders, the present study offers an insight into the evolutionary history of *UCHL1* through evolutionary rate analysis and phylogenetic investigation. Furthermore, the present study provides the comparative analysis of UCHL1 protein at sequence, structural and interaction level. Comparative sequence and structural information inferred the strong epistasis influence of evolutionary and disease causing amino-acid substitutions on a small protein segment (amino acids 32 to 39) within N-terminal C-12 peptidase domain of UCHL1. Functional implications of identified critical protein segment were further evaluated through interaction analysis of UCHL1 with its interacting partners SNCA (PARK1) and PARKIN (PARK2).

## Results

### Phylogenetic analysis

**Fig. 1:**
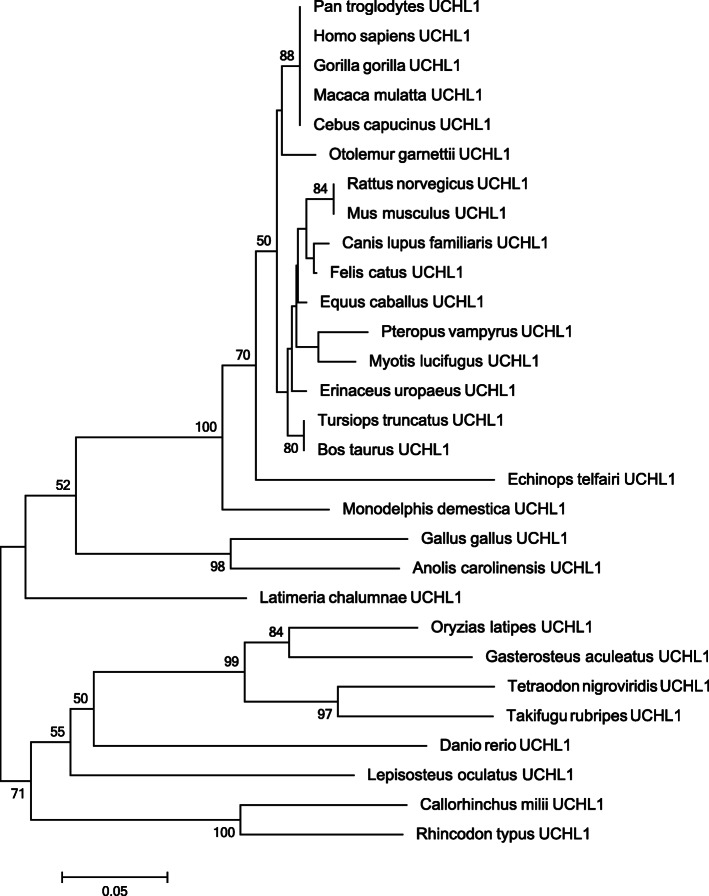
Neighbor Joining tree of UCHL1. Uncorrected p-distance and the Complete-deletion option were used. Numbers on branches represent bootstrap values (based on 1000 replications) supporting that branch; only the values ≥50% are presented here. Scale bar shows amino acid substitution per site

In order to analyze the evolutionary history of PD-associated *UCHL1* gene, Neighbor-joining (NJ) and Maximum likelihood (ML) trees were constructed. The phylogenetic tree topology suggests that *UCHL1* is a gnathostomata (jawed vertebrate) specific gene and present in tetrapods, bony fishes and in cartilaginous fishes. Bidirectional BLAST based similarity searches did not identify orthologous counter part of this gene in any of the non-gnathostomata vertebrates or invertebrate animals analyzed in the present study. Furthermore, similarity searches failed to detect any paralogous copy of *UCHL1* in any of the gnathostomata clades analyzed. (Fig. 1 and Additional file 1: Figure S1).

### Evolutionary rate analysis of *UCHL1* gene in sarcopterygians

In order to analyze evolutionary rate differences of the *UCHL1* gene within different groups of sarcopterygians lineage, key animals were selected from four groups like hominoids (human, chimpanzee, gorilla and orangutan), non-hominoids (macaque, squirrel monkey, marmoset and Otolemur), non-primate placental mammals (cow, cat, elephant and mouse) and non-mammalian tetrapods (chicken, zebra finch, turtle and coelacanth). The rationale behind choosing only sarcopterygians for evolutionary rate analysis is that animals of this lineage are known to be more closely related or show more homology to humans as compare to teleost and cartilaginous fishes [24]. To evaluate the selection constraints on selected subgroups of animals, the rates of non-synonymous (Ka/dN) and synonymous (Ks/dS) substitutions were estimated and their difference was calculated using z-test [25]. In general, Ka value lower than Ks (Ka < Ks) suggests negative selection, i.e. non silent substitutions have been purged by natural selection, whereas the inverse scenario (Ka > Ks) implies positive selection, i.e. advantageous mutations have accumulated during the course of evolution. However, the evidence for positive or negative selection requires the values to be significantly different from each other [26, 27].

The Ka-Ks (dN-dS) difference was − 2.747 (*P* = 0.007) for hominoids, − 6.501 (P = 0) for non-hominoids, − 8.945 (P = 0) for non-primate placental mammals, − 8.199 (P = 0) for non-mammalian tetrapods (Additional file 2: Table S1). These data suggest that during the course of sarcopterygians evolution, *UCHL1* has deviated significantly from neutrality and evolved under strong negative selection (Additional file 2: Table S1).

### Domain organization of UCHL1 protein

**Fig. 2:**
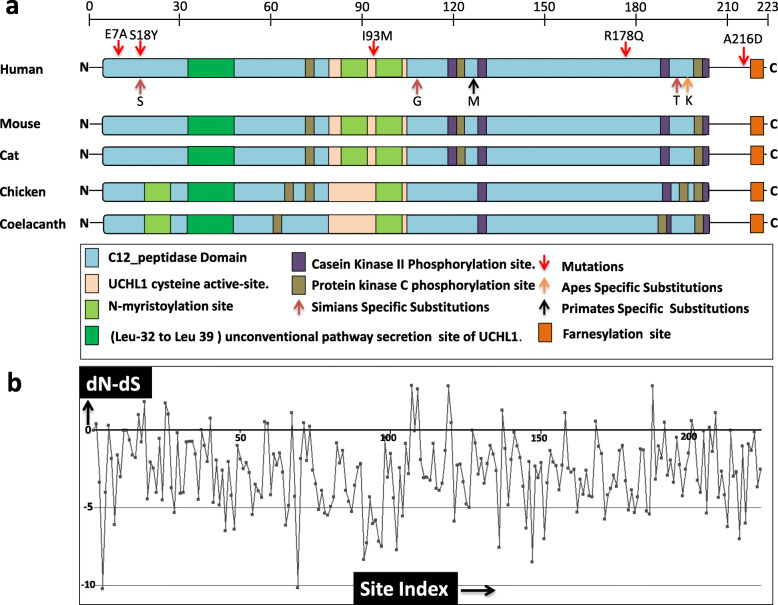
Domains organization of UCHL1 protein. **a** Diagrammatic representation of UCHL1 domains and motifs. Schematic view of comparative organization of domains and motifs of UCHL1 orthologous proteins from phylogenetically distant species. Protein and domain lengths are drawn approximately to scale. Domains and motifs are color coded. Numbers on the scale represents amino acids. **b** Plot depicts those amino acid sites of UCHL1 which are under negative selection constraint in sarcopterygians. X-axis depicts site index of each residue of UCHL1 and y-axis depicts dN-dS rate analysis of each amino acid residue of UCHL1

In order to investigate the comparative domain organization of UCHL1, complete domain, motifs and sub-motifs were identified through extensive literature survey as well as using different tools/databases [28, 29]. UCHL1 contains a single domain that spans a large portion of protein (amino-acids 3 to 206), which is named as C-12 peptidase domain. It regulates substrate access to the catalytic site [30]. The C-12 peptidase domain comprises of cysteine active-site (84–100)**,** N-myristoylation sites (87–92, 94–99), Casein Kinase II Phosphorylation sites (119–122, 125–128, 188–191, 205–208)**,** Protein kinase C phosphorylation sites (76–78, 121–123, 205–207) [29] and unconventional pathway secretion site of UCHL1 (32–39) [31]. The farnesylation-site (220–223) [32] resides outside of the C-12 domain at the C-terminal of UCHL1 (Fig. 2a). These domains, motifs and sub-motifs are comparatively mapped on orthologs from major representative species of sarcopterygians, like primate (human), non-primate placental mammals (mouse and cat), non-mammalian tetrapods (chicken) and lobe-finned fish (coelacanth) (Fig. 2a).

Annotation and comparative analyses at sequence level revealed that the C-12 peptidase domain and its major motifs like the unconventional pathway secretion site of UCHL1 (32–39) [31], cysteine active-site (84–100) and N-myristoylation sites (87–92, 94–99) are highly conserved within selected subgroup of sarcopterygians animals (Fig. 2a). Three protein kinase C (PKC) phosphorylation sites (76–78, 121–123, 205–207) and four casein kinase II (CK2) phosphorylation sites (119–122, 125–128, 188–191, 205–208) are also found to be highly conserved in mammals (human, mouse and cat), whereas in chicken a PKC phosphorylation site appears to have been translocated from C-terminal to N-terminal of UCHL1 and an additional PKC phosphorylation site (198–200) is detected. Furthermore, both in chicken and coelacanth one N-myristoylation site appears to have been translocated from C-terminal to N-terminal of UCHL1 (at position 21–26). In both chicken and coelacanth, a CK2 phosphorylation site (119–122) is not detected and some translocations of PKC phosphorylation sites are also observed (Fig. 2a).

By employing ancestral reconstruction technique five mammalian specific evolutionary substitutions were identified and mapped on human UCHL1 protein. One of them occurred at the root of primate’s lineage, three at the root of simian’s lineage and one specifically in ape’s history (Fig. 2a and Table 1). Analysis of physicochemical properties of these five mammalian specific amino acid substitutions suggests that all of them are of radical type (Table 1). All these evolutionary substitutions are localized within the C-12 peptidase domain (Fig. 2a). In addition, previously reported 5 PD and other neurological disorders associated missense mutations (E7A, S18Y, I93M, R178Q and A216D) are also localized to the C-12 peptidase domain except A216D which resides at the C-terminal of human UCHL1. These data reveal that highly conserved C-12 peptidase domain has a significant role not only in the evolutionary/functional perspective but also in disease pathogenesis. The SLAC-window analysis identified 73 negatively constrained sites within the C-12 peptidase domain of UCHL1 (Fig. 2b and Additional file 2: Table S2).

**Table 1:** Amino-acid replacements in UCHL1 during primate history

Position	Mammals ancestral residues	Replacement in ancestor of primates	Replacement in ancestor of simians	Replacement in ancestor of apes.	Neutral/Radical	Impact on protein stability	Location motif/domain
18	A		S		Radical (+ 1)	Decreased (−0.85)	Peptidase C12 domain
107	E		G		Radical (−2)	Decreased (−1.79)	Peptidase C12 domain
124	L	M			Radical (+ 2)	Decreased (−0.74)	Peptidase C12 domain
192	S		T		Radical (+ 1)	Decreased (−0.79)	Peptidase C12 domain
195	Q			K	Radical (+ 1)	Decreased (−1.45)	Peptidase C12 domain

After the divergence from mammal’s ancestor, one amino-acid change occurred in the root of primates, three replacements occurred specifically in the ancestor of Simians lineage whereas one replacement occurred in apes history. The 6th column depicts the putative physicochemical impact of each replacement on protein/structure function. The number within brackets is the log odds associated with changing the amino acids. Positive numbers imply a preferred change, zero implies a neutral change and negative numbers imply an un-preferred change. The 7th column depicts the putative impact of each replacement on stability of protein. The numbers within brackets are the confidence scores (i.e. from −1 to 1) associated with the impact on stability of protein structure by changing the amino acids. Positive numbers imply an increase in stability of protein structure. The bigger the score, the more confident the prediction is. Conversely, negative numbers imply a decrease in the stability of protein structure

### Protein structural evolution of UCHL1

**Fig. 3:**
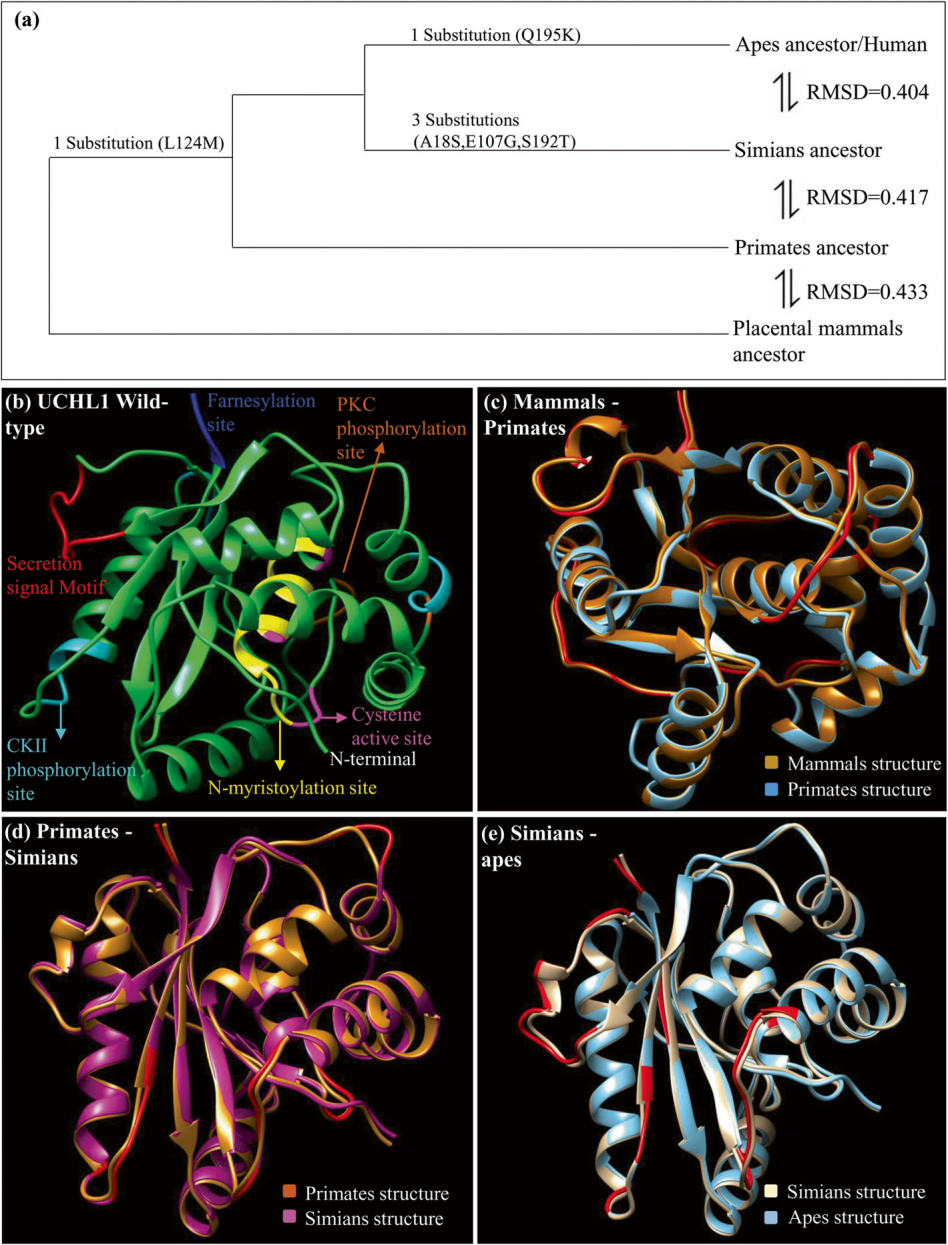
Structural evolution of UCHL1 protein. **a** Structural divergence of human UCHL1 after the split from placental mammals common ancestor. One primate specific substitution has occurred which is retained in simians and apes after they diverged from the placental mammalian ancestor. Whereas, three substitutions have occurred specifically at the root of simians lineage and one apes specific substitution is also detected. Structural deviations among ancestors are examined by RMSD values. **b** Annotated structure of wild type UCHL1. **c** Superimposed placental mammals ancestor and primates ancestor structures. **d** Superimposed primates ancestor and simians ancestor structures. **e** Superimposed simians ancestor and apes ancestor/human structures. Deviated residues in terms of backbone torsion angles (Φ°, Ψ°) are represented in red color and all superimposed structures are color coded

Comparative protein structure modeling was performed to inspect how negative selection is performing its role in defining the spatial constrains on ancestral UCHL1 proteins. Ancestral protein structures (mammals, primates, simians and apes) obtained from the MODELLER program were superimposed at an appropriate evolutionary scope, i.e. mammals-primates ancestors, primates-simians ancestors and simians-apes/human ancestors (Fig. 3). Protein structural deviations were examined with the help of Chimera and root mean square deviation (RMSD) values (Fig. 3 and Table 2). Comparative structural investigations revealed very notable aspects of UCHL1 evolution that were not anticipated by comparative analysis at sequence level alone. It appears that during the course of mammalian evolution the UCHL1 has undergone strong intragenic epistatic interactions to acquire its favorable protein conformation. Aforementioned superimposed protein models revealed a common deviated region composed of amino acids 32 to 39 within the secretion site at the N-terminal of C-12 peptidase domain (Table 2). These structural deviations were also measured with the help of backbone torsions quantification which also suggests that protein segment composing of amino acids 32 to 39 has evolved during mammalian history through a well-known phenomenon called intragenic epistasis [33]. It appears that during the course of evolution, mammalian UCHL1 has incorporated destabilizing substitutions to obtain its intrinsic disordered conformation through radical structural shifts in this identified critical region. This critical region (amino acids 32–39) is recognised as crucial for proper conformation of not only unconventional pathway secretion site of UCHL1 protein but also for the entire C-12 peptidase domain.

**Table 2:** Structural comparisons of predicted ancestral UCHL1 proteins

Comparison between lineages	Major change in backbone torsion angles	Major shifts in region	Critical region
Mammals Primates ancestor	Leu32-Gly40 Pro43-Ala46, Lys71-Ser76, Gln103-Gly111, Asn136, Glu149-Arg153, Gly210-Valn212	Secretion Signal motif C12- Peptidase domain C-terminal region	32–39 (Secretion Signal Motif)
Primates Simians ancestor	Ser41-Val43, Ala48-Leu50, Gln84-Gly87, Glu109-Gly111, Gly150-Asp156, Gly210-Ser215, Ala222-Ala223	C12-peptidase Domain C-Terminal region Farnesylation site	___
Simians Apes/human ancestor	Val31-Gly39 Ser41-Val42, Glu208-Ser215, Glu149-Cys152, Val154-Asn159, Glu203	Secretion Signal motif C12- Peptidase domain C Terminal region	32–39 (Secretion Signal Motif)

This table shows the effect of lineage specific substitutions on the backbone torsion angles of ancestral UCHL1 protein structures through comparisons among lineages given in first column

**Fig. 4:**
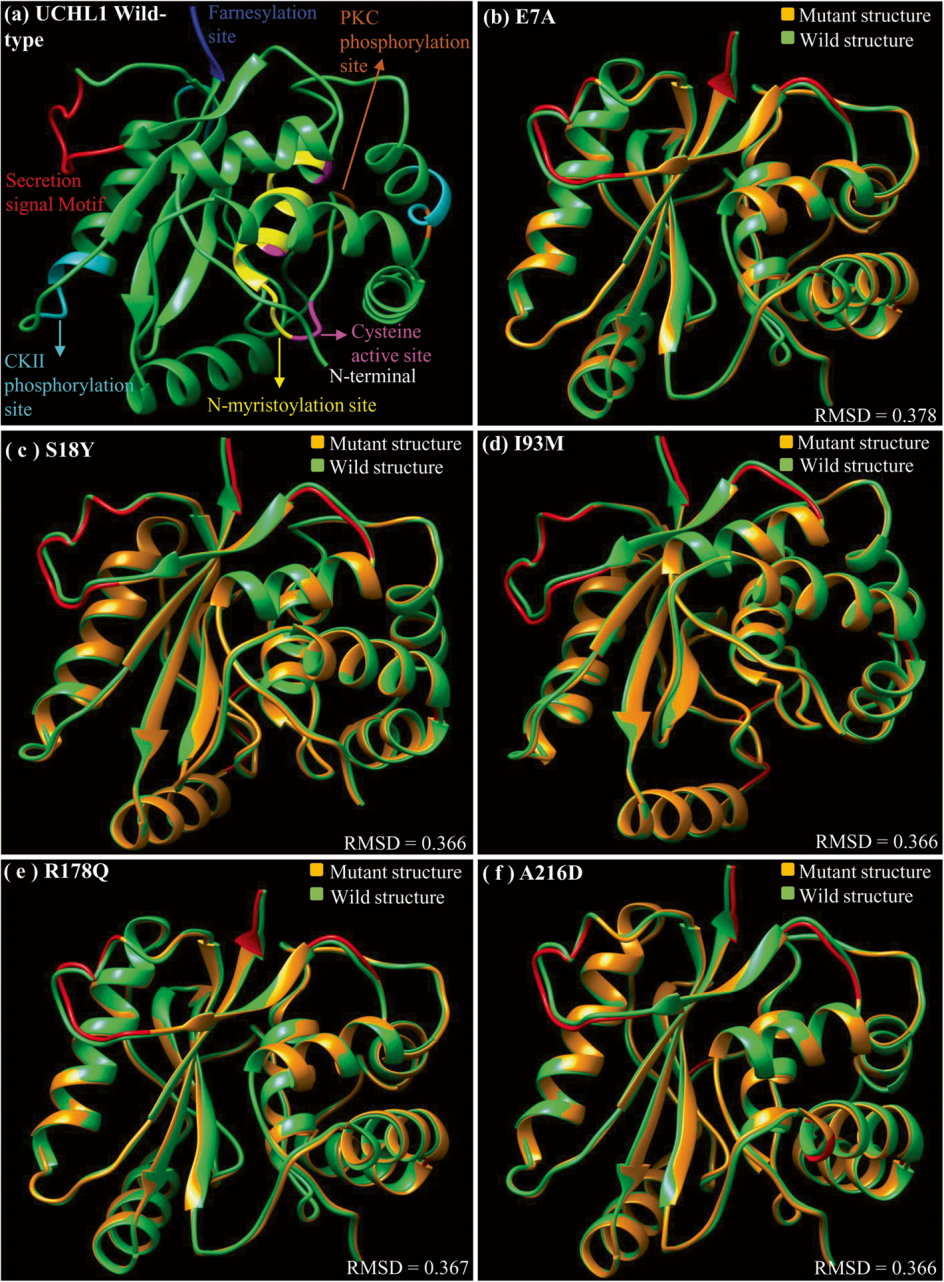
Protein structural deviations in PD associated mutant versions of UCHL1. Major structural shifts caused by disease associated missense mutations of UCHL1 are observed in the secretion signal and farnesylation motifs present in the N-terminal and C-terminal respectively. Deviated residues are labeled in red. **a** Structure of wild type UCHL1 in which all domains and motifs are color coded. **b** Structural superimposition of wild type (green) and mutated model E7A (coral peach). **c** Structural comparison between wild type UCHL1 (green) and mutated model S18Y (coral peach). **d** Structural deviations among wild type UCHL1 (green) and mutated model I93M (coral peach). **e** Structural comparison between UCHL1 (green) and mutated model R178Q (coral peach). **f** Structural superimposition of the wild type (green) and mutated model A216D (coral peach)

Interestingly, all the previously reported human specific missense mutations associated with familial and sporadic PD and other neurological disorders are confined to the C-12 peptidase domain except A216D. To investigate the impact of these previously reported missense mutations at protein structure level, all disease causing mutant structures (previously reported missense mutations) of UCHL1 were predicted through MODELLER and superimposed on 2ETL (the wild type version of UCHL1) (Fig. 4). In addition, wild-type protein structure of UCHL1 and disease causing mutant versions were also predicted through I-TASSER server (Additional file 1: Figure S2 and Additional file 2: Table S3) and Robetta server (Additional file 1: Figure S3 and Additional file 2: Table S4). I-TASSER and Robetta predicted wild-type and mutant protein structures of UCHL1 were also superimposed (Additional file 1: Fig. S2, Fig. S3 and Additional file 2: Table S3, Table S4). Structural deviations between wild type and mutant versions were evaluated using RMSD values. Intriguingly, even though the disease causing mutations are spread across UCHL1, all of them appear to impact the structure of common protein region spanning amino acids 32–39 (secretion site of UCHL1). In addition another protein region (amino acids 222–223) within farnesylation site is deviated in all disease causing mutant models except for E7A (Fig. 4 and Table 3; Additional file 1: Figure S2 and Figure S3; Additional file 2: Table S3 and Table S4). Therefore, UCHL1 protein segment spanning amino acids 32–39 is considered as critical not only in evolutionary perspective but also in disease pathogenesis.

**Table 3:** Structural comparisons of disease causing mutant versions of UCHL1 protein

Mutations	Major changes in residue number	Major shifts in region	Critical region
Ile93Met	23–24	C12-peptidase Domain	32–39 (Secretion, Signal Motif)
	32–38	Secretion signal Motif	
	45,74–75		
	136,148		
	222–223	Farnesylation site	
Glu7Ala	22–25	C12- peptidase Domain	32–39(Secretion, Signal Motif)
	31–38	Secretion signal Motif	
	45,74–75		
	111–112		
	190–191,136	CK2 Phosphorylation site	
Ser18Tyr	23–25,	C12 peptidase Domain	32–39 (Secretion, Signal Motif)
	31–38,	Secretion signal Motif	
	45,74–75		
	111–112		
	150–152		
	148,136		
	222–223	Farnesylation site	
Arg178Gln	23–24	C12-peptidase Domain	32–39(Secretion, Signal Motif
	32–39	Secretion signal Motif	
	136,74		
	221–223	Farnesylation site	
Ala216Asp	23–24	C12-peptidase Domain	
	74–75		___
	136,148		
	222–223	Farnesylation site	

This table shows the impact of PD (Parkinson’s disease) and other neurological disorders causing missense mutations on backbone torsion angles of human UCHL1 protein. In the first column, amino acid residue on the left indicates the wild-type residue, whereas the residue on the right is mutated version; number indicates the amino acid position. The second column specifies the positions at which major structural deviations are observed. The third column depicts the deviated region. The fourth column depicts deviated residues shared among all mutant structures of UCHL1 protein

### Protein-protein interaction analysis of UCHL1

**Fig. 5:**
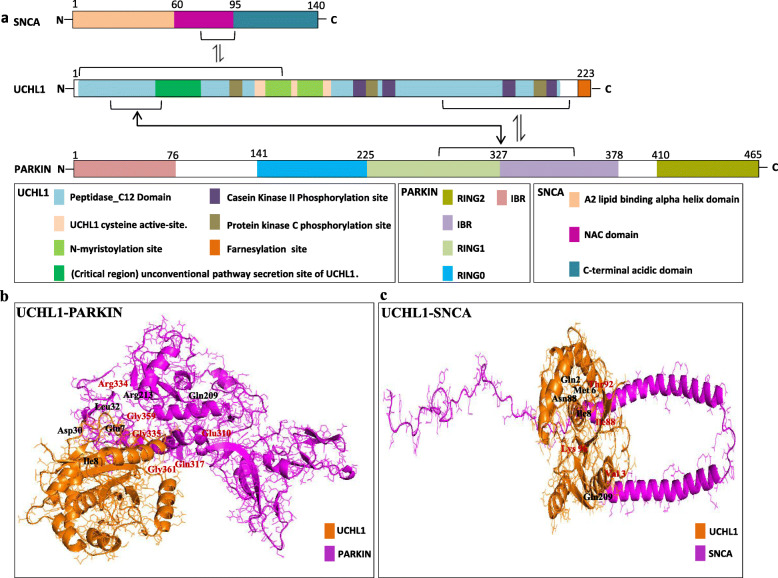
Comparative domain organization of PD causing proteins and their interacting residues. **a** Comparative organization of key functional domains and motifs of human UCHL1, SNCA and PARKIN. Protein lengths are drawn approximately to scale. Domains and motifs are color coded. **b** Docked complex and hydrogen bonds between human UCHL1 (2ETL) and human PARKIN proteins. Interaction structures are color coded. Total 15 interacting residues are identified, subset of them are displayed on the docked complex. UCHL1 interaction residues are shown in black color, whereas PARKIN residues are shown in red color. **c** Docked complex and hydrogen bonds between human UCHL1 (2ETL) and human SNCA proteins. Interaction structures are color coded. Total 5 interacting residues are identified and all of them are displayed on the docked complex. UCHL1 interaction residues are shown in black color whereas SNCA residues are shown in red color

In order to further investigate the significance of identified critical protein segment, we performed the interaction analysis of human UCHL1 with its biochemically and genetically verified interacting partner proteins. The identification of pathogenic mutations in the three human genes, i.e. SNCA (PARK1), PARKIN (PARK2), and UCHL1 (PARK5) has elucidated the ubiquitin proteasome system (UPS) and its potential role as a causal pathway in PD [34]. Furthermore, the STRING database reveals information that SNCA and PARKIN are the interacting partners of UCHL1 [35]. For protein-protein interaction analyses the domain architecture of SNCA and PARKIN is also annotated (Fig. 5). Human SNCA protein comprises of 140 amino acids and contains three major domains named as A2 lipid binding alpha helix domain, NAC domain and C-terminal acidic domain [2]. Furthermore, PARKIN comprises of 465 amino acids and contains 5 domains named as Ubl domain, RING0, RING1, in-between RING (IBR) domain and RING2 domain [36].

Interaction analysis between UCHL1 and SNCA depicts only five interacting residues that involves the C-12 peptidase domain of UCHL1 and the NAC domain of SNCA (Fig. 5 and Additional file 2: Table S5). Interaction analysis of five human disease causing mutant versions of UCHL1 with SNCA revealed altered interaction patterns, although some of the wild type interactions are also retained (Additional file 1: Fig. S4 and Additional file 2: Table S6). These data signifies that UCHL1 and SNCA not only interact in normal individuals but also in PD patients with altered interaction pattern (Fig. 5; Additional file 1: Figure S4 and Additional file 2: Table S6).

Intriguingly, in UCHL1 (R178Q)-SNCA docked complex, two altered interactions were found within the identified critical region (amino acids 32–39 within secretion site) of UCHL1, which could potentially affect the normal secretion of UCHL1 to neurons and thus could contributes to disease pathogenesis (Additional file 1: Figure S4 and Additional file 2: Table S6).

Interaction analysis of UCHL1 with PARKIN showed 15 interacting residues involving RING1 and IBR domains of PARKIN and C-12 peptidase domain of UCHL1 (Fig. 5 and Additional file 2: Table S5).

## Discussion

Advent of high throughput annotation of genomes and broadened availability of genomic sequence data permitted to investigate the evolutionary history of genes of interest and link it to human disease associated phenotypic traits [26]. Ubiquitin proteasome pathway (UPP) is the most important molecular mechanism that participates in neurodegenerative diseases such as PD and AD. The major pathological hallmarks of these two important neurodegenerative disorders are characterized by the accumulation of abnormal protein aggregates (AD: Extracellular Aβ amyloid plaque; PD: Intracellular α-synuclein in the Lewy body) within the neurons [37, 38]. UPP is the major mechanism which serves to recognize the damaged or misfolded proteins and transport them to the proteasome for degradation. By this way UPP maintains the normal concentration of proteins in the neurons and thus prevent neurodegeneration [39, 40]. UPP contains several components that include 26S proteasome, ubiquitin, ubiquitin activating enzyme E1, ubiquitin conjugating enzyme E2 and ubiquitin ligating enzyme (E3). The *UCHL1* gene product is a key component of UPP and functions as deubiquitinating enzymes to remove ubiquitin from proteins and it also stabilizes the monomer ubiquitin in cell free system [11, 41]. Furthermore, UCHL1 is also known to facilitate E3 ligase in UPP [42–44]. The *UCHL1* gene product has also been implicated in processes like apoptosis, protection against oxidative stress [45], long-term protonation [20] and chaperone-mediated autophagy [16]. However, the role of UCHL1 in these processes have not yet been fully understood [32].

UCHL1 consists of 223 amino acids with a single domain. It belongs to a α/β fold protein family with six β-strands inside the hydrophobic core, which is surrounded by seven α-helices [12]. Five missense mutations in human *UCHL1* gene have so far been associated with autosomal dominant PD, recessive hereditary spastic paraplegia (SPG79) and early onset of progressive neurodegeneration. The present study is an attempt to investigate sequence and structural bases of UCHL1 evolution within the sarcopterygians lineage. Furthermore, this study elucidates protein structural and interactional basis of UCHL1-associated PD pathogenesis.

The ML and NJ based phylogenetic tree topologies of the *UCHL1* gene supported by high bootstrap values revealed that it is gnathostomata (jawed-vertebrate) specific gene and present in tetrapods, bony fishes and in cartilaginous fishes. Bidirectional BLAST based similarity searches did not identify orthologous counter part of this gene in any of the non-gnathostomata vertebrates and invertebrate animals analyzed. Furthermore, similarity searches failed to detect any paralogous copy of *UCHL1* in any of the gnathostomata (jawed-vertebrate) clades analyzed. Based on these phylogenetic data, it is speculated that human *UCHL1* might have originated at the root of jawed vertebrates (Fig. 1 and Additional file 1: Figure S1). These observations prompted us to evaluate the selection constraints on this gene within different selected subgroups of animals in sarcopterygians lineage. For this purpose, the rates of non-synonymous (Ka/dN) and synonymous (Ks/dS) substitutions were estimated within each selected subgroups of animals and their difference was calculated using z-test. These statistical estimations corroborate well with the speculation that *UCHL1* has evolved under strong purifying selection throughout the sarcopterygians history, which might have discouraged any functional modification to happen through gene duplications (Additional file 2: Table S1).

The C-12 peptidase domain is a major domain that spans the large portion of UCHL1 starting from residue 3 (at N-terminal) and ending at 206 residues (at C-terminal). Comparative domain organization data suggests that the C-12 domain and its motifs/sub-motifs are similarly present in all of the animals analyzed with no major variations in protein primary organization. In addition to C12 peptidase domain, extreme N-terminal and C-terminal sites also appear to be highly preserved among all the analyzed sarcopterygian animals (Fig. 2). Intriguingly, conserved domain/motif organization of UCHL1 among distantly related orthologs corroborates well with the signal of strong purifying selection identified through z-statistics and with previously reported functional data on human UCHL1. For instance, a recent study suggests that removal of only eleven residues from the N-terminal of UCHL1 is sufficient for the protein to loss affinity for ubiquitin or lack of deubiquitinating activity and ultimately leads to formation of insoluble aggregates [46, 47]. Minor truncation at N- or C-terminus of UCHL1 are reported to denature the protein, therefore renders it functionless [48]. In vitro mutagenic and *in-silico* simulation studies revealed that removal of few amino acids either from the C-terminal or from the N-terminal of UCHL1 can destabilize its three dimensional structure, resulting in unfolding or loss of solubility consistent with protein aggregation [46, 48, 49].

Comparative structural analyses were performed to inspect how negative selection is playing its role in defining the spatial constrains on ancestral UCHL1 at structural level (Fig. 3 and Table 2). The comparative structural analysis of predicted ancestral proteins of UCHL1 (mammals ancestor, primates ancestor, simians ancestor and apes/human ancestor) showed multiple deviated regions in each of the predicted ancestral UCHL1. Comparative analysis of ancestral predicted structural data revealed a common deviated region comprises of amino acids 32 to 39 (Fig. 3). This particular protein segment appears to have experienced structural shifts repeatedly during the course of mammalian evolution through strong intragenic epistatic interactions within UCHL1. Furthermore, the protein structural impacts of five identified lineage specific substitutions suggest that UCHL1 has undergone structural destabilization during the course of mammalian history (Table 1). This structural destabilization can best be explained by speculating that during the course of mammal’s evolution UCHL1 has acquired the ability to attain favorable conformation upon binding to its targets (Table 1).

Furthermore, we also performed comparative structural analysis of previously reported PD and other neurological disorders causing missense mutations of UCHL1 (between wild type and all five PD and other neurological disorders causing mutant versions). This comparative structural analysis (predicted through Modeller, I-TASSER and Robetta) revealed multiple distinct deviated regions in each comparison as well as a commonly deviated segment comprising of amino acids 32 to 39 within the secretion site at N-terminal of the C-12 peptidase domain (Fig. 4 and Table 3; Additional file 1: Figure S2 and Figure S3; Additional file 2: Table S3 and Table S4). Interestingly, this commonly deviated region (32 to 39) in disease causing variants of UCHL1 also appears to have evolved structurally during mammalian evolution (described in preceding sections). Therefore, the protein segment 32–39 of UCHL1 is both important and indispensable in evolution and PD pathogenesis through intraprotein conformational epistasis [33]. The functional significance of this critical region is supported by previously reported data which suggest that the substitution of the leucine within this region (Leu-32 to Leu-39) leads to reduced secretion of UCHL1 in cytoplasm of neuronal cells and consequently causing PD phenotype [31]. In addition, the comparative analysis of human UCHL1 structure with all five PD-causing mutant versions (except E7A) revealed another commonly deviated region at C-terminal comprises of protein segment 220–223 within farnesylation site (Fig. 4 and Table 3; Additional file 1: Figure S2 and Figure S3; Additional file 2: Table S3 and Table S4). Functional significance of this second commonly deviated region corroborate well with previously reported data which suggest that the loss of just four amino acids from the C-terminal of UCHL1 is sufficient to induce protein aggregation and consequently neuronal cell death [32, 49]. Taken together, critical regions of UCHL1 identified in the present study (amino acids 32–39 & amino acids 220–223) are important for maintaining normal neuronal physiology and any conformational changes in either of these protein regions could lead to PD disease.

The interaction analysis of UCHL1 with its major interacting partner’s, i.e. SNCA and PARKIN further highlights the structural, functional and disease significance of identified critical regions (amino acids 32–39). For instance, the protein-protein interaction analysis revealed that the C-12 peptidase domain of UCHL1 interacts with the NAC domain of SNCA, and the RING1 and IBR domains of PARKIN (Fig. 5). Interestingly, both SNCA and PARKIN appears to interact physically with the identified critical region (amino acids 32–39) of UCHL1 (Fig. 5 and Additional file 2: Table S5). In addition, interaction analysis of five human disease causing mutant versions of UCHL1 with SNCA revealed altered interaction pattern, although some of the wild type interactions are also retained. For instance, docked complex of wild type UCHL1 with disease-causing mutant version of SNCA (A30P) revealed no altered interactions (same as the wild type interaction of UCHL1-SNCA complex) (Additional file 1: Figure S5 and Additional file 2: Table S6). In contrast, docked complex of wild type UCHL1 with another disease-causing mutant version of SNCA (A53T) revealed altered interaction pattern (Additional file 1: Figure S5 and Additional file 2: Table S6). These interaction data corroborate well with previously reported biochemical data which suggests that the A53T mutant version of SNCA impacts the normal secretion of UCHL1 in neuronal cytoplasm [31]. Based on these interaction analyses, it is speculated that UCHL1 and SNCA interact physically, not only in normal individuals but also in PD patients with altered interaction pattern. (Fig. 5; Additional file 1: Figure S4 and Additional file 2: Table S6).

## Conclusion

Human UCHL1 is known to play an important role in ubiquitin stability within neurons which is critical for ubiquitin–proteasome system and neuronal survival. Mutations in the human *UCHL1* gene have been associated with various neurodegenerative disorders like PD, recessive hereditary spastic paraplegia (SPG79), AD and Huntington’s disease. Considering the indispensable role of the *UCHL1* gene product in neuronal physiology and pathophysiology, the current study investigates the sequence evolutionary pattern and structural dynamics of UCHL1. Phylogenetic data suggest the ancient origin of UCHL1 at the root of gnathostomes (jawed vertebrate) history. Furthermore, molecular sequence evolutionary analysis reveals that *UCHL1* has remained under strong functional constraints throughout the gnathostomes history which might have discouraged the duplication of this gene in any of the animal lineage analyzed in the present study. Comparative structural analysis of UCHL1 pinpointed a critical protein segment (amino acids 32 to 39 within the secretion site) with crucial implications in evolution and PD pathogenesis through a well known phenomenon of intraprotein conformational epistasis. This critical protein segment of UCHL1 can be targeted for drug designing and investigation for the treatment of PD in future.

## Methods

### Sequence acquisition

The putative orthologous protein sequences of human UCHL1 were retrieved from protein databases accessible at Ensemble [50] and National Center for Biotechnology Information [51] by using BLAST p bidirectional best hit approach [52]. Further confirmation of the common ancestry of the putative orthologs was obtained by clustering homologous proteins within phylogenetic trees [53, 54]. Sequences whose position within a tree is in sharp conflict with the uncontested animal phylogeny are excluded from the analysis. All protein sequences used in this study are provided in Additional file 3.

List of species selected for sequence analysis of UCHL1 is *Homo sapiens* (Human), *Pan troglodytes* (Chimpanzee), *Gorilla gorilla* (Gorilla), *Macaca mulatta* (Macaque), *Cebus capucinus* (white-faced sapajou), *Otolemur garnettii* (Northern greater galago), *Mus musculus* (Mouse), *Rattus norvegicus* (Rat), *Equus caballus* (Horse), *Tursiops truncatus* (Dolphin), *Bos taurus* (Cow), *Felis catus* (Cat), *Canis lupus familiaris* (Dog), *Pteropus vampyrus* (Megabat), *Myotis lucifugus* (Micro bat), *Erinaceus europaeus* (Hedgehog), *Echinops telfairi* (lesser hedgehog tenrec), *Monodelphis domestica* (Opossum), *Gallus gallus* (Chicken), *Anolis carolinensis* (Anole lizard), *Latimeria chalumnae* (Coelacanth), *Oryzias latipes* (Medaka), *Gasterosteus aculeatus* (Stickleback), *Tetraodon nigroviridis* (Tetraodon), *Takifugu rubripes* (Fugu), *Danio rerio* (Zebra fish), *Lepisosteus oculatus* (Spotted gar), *Callorhinchus milii* (Elephant shark), *Rhincodon typus* (Whale Shark).

### Sequence analysis

Protein sequences were aligned by using CLUSTAL W through MEGA5 [25]. The phylogenetic tree of UCHL1 was reconstructed by applying NJ method [55, 56]. Complete deletion option was used for removing gaps and missing data in the protein sequences. Poisson corrected (PC) amino acid distance and uncorrected p-distance of amino acids were used as amino acid substitution models [57]. Due to the similar results obtained with both aforementioned models only NJ tree based on uncorrected p-distance is presented (Fig. 1). ML tree was also constructed by using the Whelan and Goldman (WAG) model of amino acid substitutions [58] (Additional file 1: Figure S1). To ensure the reliability and accuracy of the both NJ and ML trees, topologies bootstrap method was used (at 1000 pseudo replicates), which assigns the bootstrap values to each branch of the tree [59].

ML method and WAG model of amino acid substitution were used to predict ancestral sequences of UCHL1. Z-test is executed with MEGA [25] to examine selection constraint within hominoids (human, chimpanzee, gorilla and orangutan), non-hominoids (macaque, marmoset, squirrel monkey and bush baby), non-primate placental mammals (mouse, cat, cow and elephant) and non-mammalian tetrapods (chicken, turtle, frog and coelacanth). Goldman And Yang (GY-94) method (codon based model) implemented in Hyphy program was employed to calculate dN-dS for each of the aforementioned sarcopterygians groups [60].

Domains, motifs and sub-motifs were assigned to human UCHL1 by ratification from different databases like pfam [28] and MyHit tool [29]. Clustal Omega [61] was employed for multiple sequence alignment to map the putative positions and locations of domains, motifs and sub-motifs on human UCHL1 and also in orthologous sequences from selected sarcopterygian animals (Fig. 2a). Identified evolutionary substitutions and previously reported human PD and SPG79 causing missense mutations (E7A, S18Y, I93M, R178Q, A216D) were mapped on the human UCHL1 (Fig. 2a). To estimate the negatively constrained residues of UCHL1 among sarcopterygians, we employed Single Likelihood Ancestor Counting (SLAC) method through Hyphy which uses global codon model and maximum likelihood to reconstruct the evolutionary history [60]. The impact of all substitutions identified within mammalian history of UCHL1 were also classified into neutral or radical on the basis of their physicochemical properties, i.e. charge, volume, polarity [62, 63].

### Structural analysis

X-ray structure of human UCHL1 (2ETL) was retrieved from RCSB Protein Data Bank (PDB) [64]. This X-ray structure was used as a reference in the comparative structural analysis to evaluate the structural deviations, both in evolutionary and disease perspective. Ancestral protein sequences (Mammalian ancestral, primate’s ancestral, simian ancestor and apes/human ancestor) were predicted by ancestral reconstruction technique with wild type UCHL1 structure as a reference to model the ancestral proteins (aforementioned) through homology modeling program MODELLER9 [65]. Best structures were scrutinized on the basis of Discrete Optimized Protein Energy score. For improving the quality of the modeled protein structures, energy minimization protocols were employed through YASARA energy minimization server [66]. For further quality validation of the modeled structures, RAMPAGE [67] and ERRAT [68] were employed (Additional file 1: Figure S6). MuPro was used to investigate the impact of lineage specific substitutions on the modeled ancestral UCHL1 [69]. X-ray structure of human UCHL1 was also used as a reference to model the protein structures of human PD and other neurological disorders causing missense variants of UCHL1 (E7A, S18Y, I93M, R178Q, and A216D) via homology modeling program MODELLER9 [65]. Furthermore, we also predicted the structures of wild-type and disease causing mutant versions of UCHL1 protein through I-TASSER server [70] (Additional file 1: Figure S3) and Robetta server (https://robetta.bakerlab.org/) (Additional file 1: Figure S4). Aforementioned protocol is used to minimize, validate and check the quality of PD and other neurological disorder causing mutant models of UCHL1 (Additional file 1: Figure S7). Superimposition of all modeled disease-causing mutant versions of UCHL1 with their wild type version was carried out by Chimera and root mean square deviation (RMSD) values were calculated [71]. For protein-protein interaction analysis of human UCHL1, Cluspro protein-protein docking server [72] was utilized. X-ray structure of interacting partners of UCHL1, i.e. SNCA and PRKIN were obtained from PDB. Interaction between human UCHL1, human SNCA and human PRKIN were examined with the help of Ligplot [73] and PyMol [74].

## Supplementary information

**Additional file 1.** Supplementary Figures.

**Additional file 2.** Supplementary Tables.

**Additional file 3.** Complete list of protein sequences used in this study.

## Data Availability

The datasets analyzed during the current study are available in the Ensemble database (http://www.ensembl.org), NCBI database (https:// www.ncbi.nlm.nih.gov/).

## Abbreviations

UCHL1: Ubiquitin C-Terminal Hydrolase 1
AD: Alzheimer’s disease
ML: Maximum Likelihood
NJ: Neighbor-Joining
PC: Poisson corrected
WAG: Whelan and Goldman
RMSD: Root Mean Square Deviation
PD: Parkinson’s disease
SLAC: Single Likelihood Ancestor Counting
PDB: Protein Data Bank
PKC: Protein Kinase C
CK2: Casein Kinase II
UPP: Ubiquitin Proteasome Pathway
