# Supplementary material for: Molecular evolutionary and structural analysis of human UCHL1 gene demonstrates the relevant role of intragenic epistasis in Parkinson’s disease and other neurological disorders
Source: BMC Evol Biol. 2020 Oct 7;20:130. doi: 10.1186/s12862-020-01684-7 (PMC7542113; doi:10.1186/s12862-020-01684-7)
Supplement: Supplementary file 1 — Additional file 1. Supplementary Figures. [file 12862_2020_1684_MOESM1_ESM.pdf]

Additional file 1 for: Molecular evolutionary and structural analysis of human *UCHL1* gene demonstrates the relevant role of intragenic epistasis in Parkinson's disease and other neurological disorders

Muhammad Saqib Nawaz[1], Razia Asghar[1] , Nashaiman Pervaiz[1], Shahid Ali[1], Irfan Hussain[1], Peiqi Xing[2], Yiming Bao[2]* & Amir Ali Abbasi[1]*

1 National Center for Bioinformatics, Program of Comparative and Evolutionary Genomics, Faculty of Biological Sciences, Quaid-i-Azam University, Islamabad 45320, Pakistan

2 National Genomics Data Center; BIG Data Center & CAS Key Laboratory of Genome Sciences and Information, Beijing Institute of Genomics, Chinese Academy of Sciences, Beijing, 100101, China

*Corresponding authors

Y.B*: E-mail: baoym@big.ac.cn

A.A.A*:E-mail:abbasiam@qau.edu.pk

Additional file 1: Supplementary Figures

Supplementary Figure S1

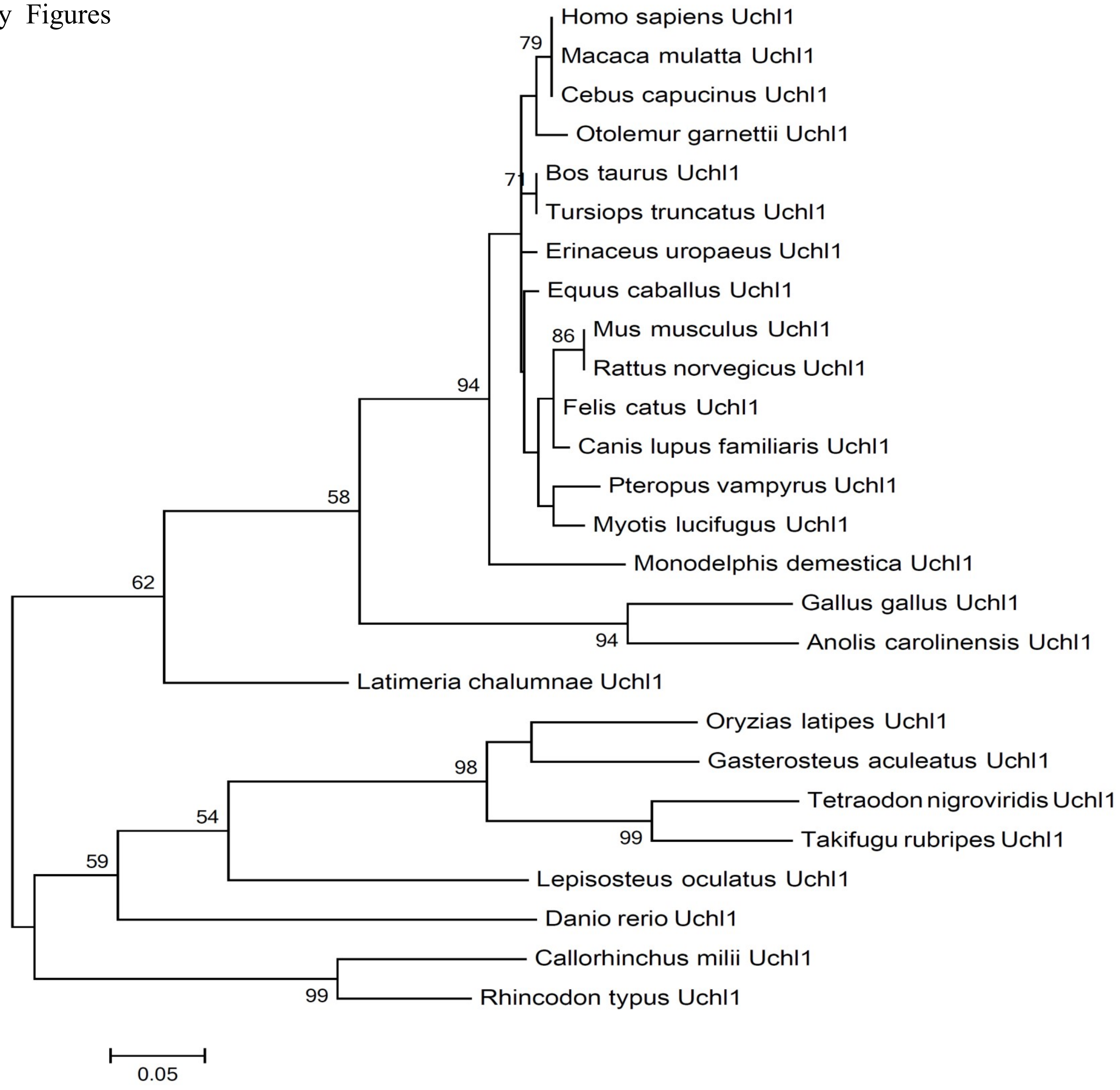

Figure S1.The evolutionary history was inferred by using the Maximum Likelihood method based on the Whelan And Goldman model. The tree with the highest log likelihood (-1749.9967) is shown. The percentage of trees in which the associated taxa clustered together is shown next to the branches. Initial tree(s) for the heuristic search were obtained automatically as follows. When the number of common sites was < 100 or less than one fourth of the total number of sites, the maximum parsimony method was used; otherwise BIONJ method with MCL distance matrix was used. The tree is drawn to scale, with branch lengths measured in the number of substitutions per site. The analysis involved 26 amino acid sequences. All positions containing gaps and missing data were eliminated. There were a total of 122 positions in the final dataset. Evolutionary analyses were conducted in MEGA5.

Supplementary Figure S2

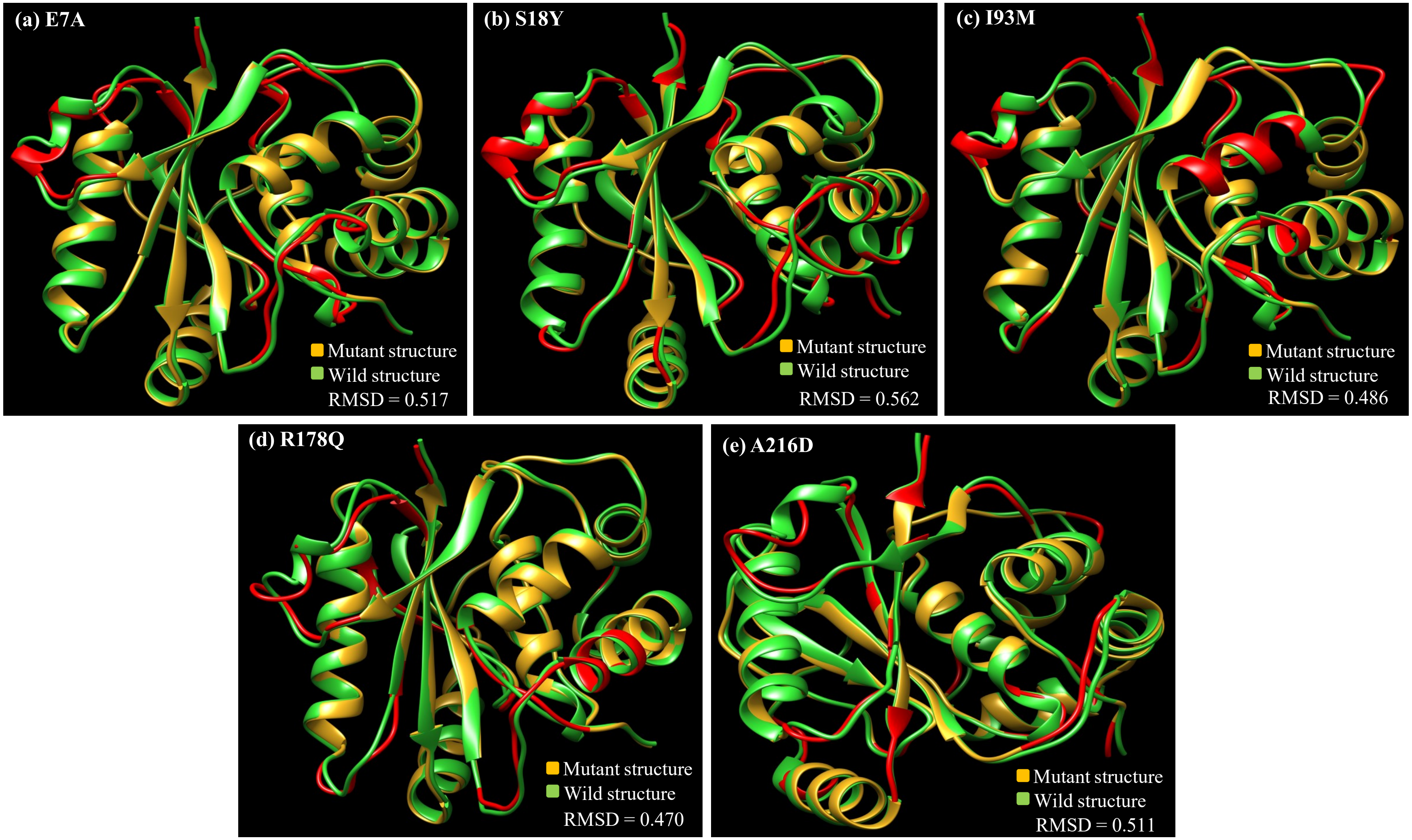

Figure S2. Structural deviations between human wild type and PD and other neurological disorders causing mutant versions of UCHL1 protein (Predicted through I-TASSER server). Major structural shifts caused by disease associated missense mutations of the UCHL1 protein are observed in the secretion signal and farnesylation motifs present in the N-terminal and C-terminal respectively. Mutated residues are labeled in red. **(a)** Shows structural superposition of wild type (green) and mutated model E7A (coral peach).**(b)** Depicts structural comparison between wild type UCHL1 (green) and mutated model S18Y (coral peach) **(c)** Represents the structural deviations among wild type UCHL1 (green) and mutated model I93M (coral peach).**(d)** Structural comparison between UCHL1 (green) and mutated model R178Q (coral peach). **(e)** Structural superimposition of the wild type (green) and mutated model A216D (coral peach).

Supplementary Figure S3

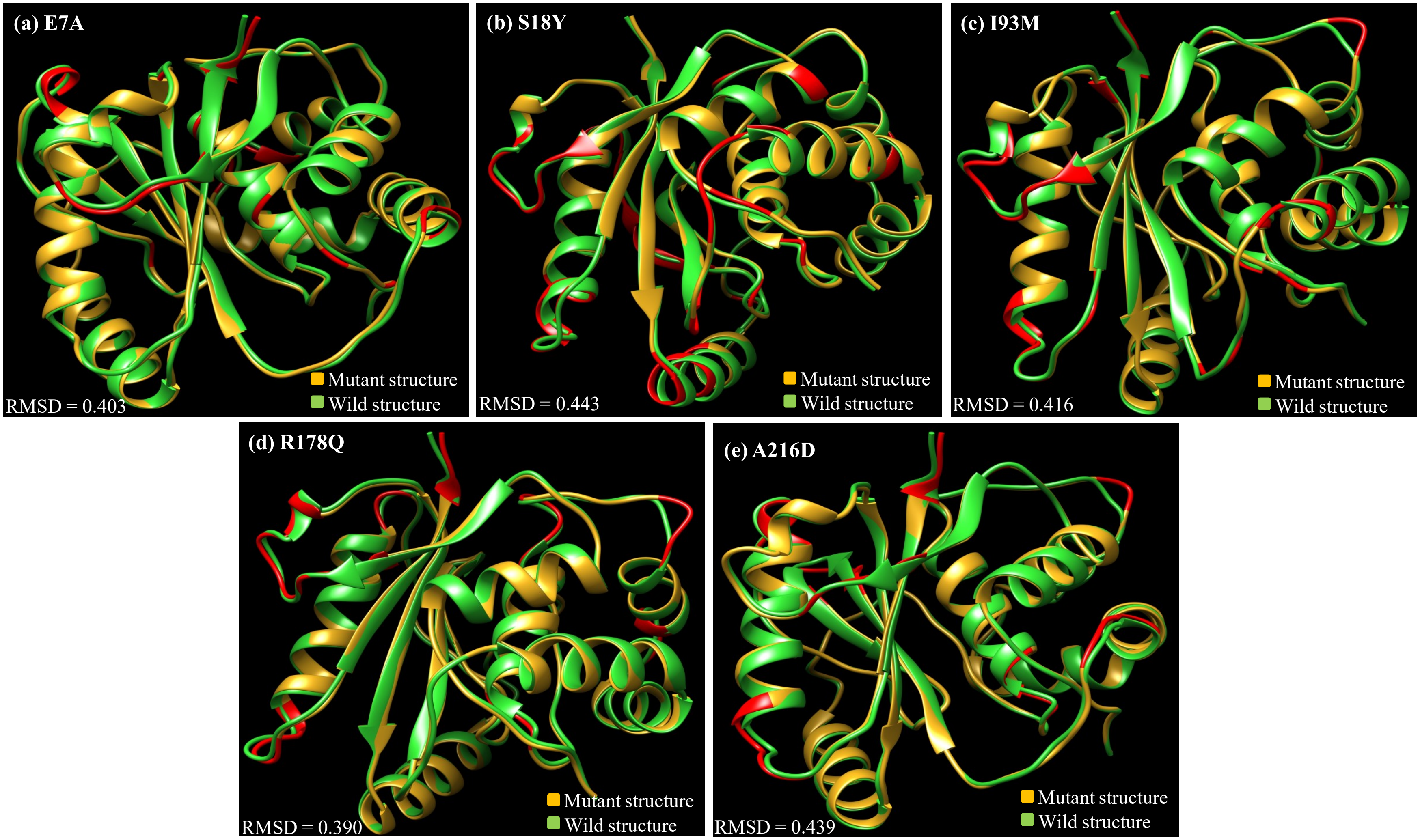

Figure S3. Structural deviations between human wild type and PD and other neurological disorders causing mutant versions of UCHL1 protein (Predicted through Robetta server). Major structural shifts caused by disease associated missense mutations of the UCHL1 protein are observed in the secretion signal and farnesylation motifs present in the N-terminal and C-terminal respectively. Mutated residues are labeled in red. **(a)** Shows structural superposition of wild type (green) and mutated model E7A (coral peach).**(b)** Depicts structural comparison between wild type UCHL1 (green) and mutated model S18Y (coral peach) **(c)** Represents the structural deviations among wild type UCHL1 (green) and mutated model I93M (coral peach).**(d)** Structural comparison between UCHL1 (green) and mutated model R178Q (coral peach). **(e)** Structural superimposition of the wild type (green) and mutated model A216D (coral peach).

Supplementary Figure S4

(a) UCHL1 Mutant (E7A) and SNCA wild type Complex

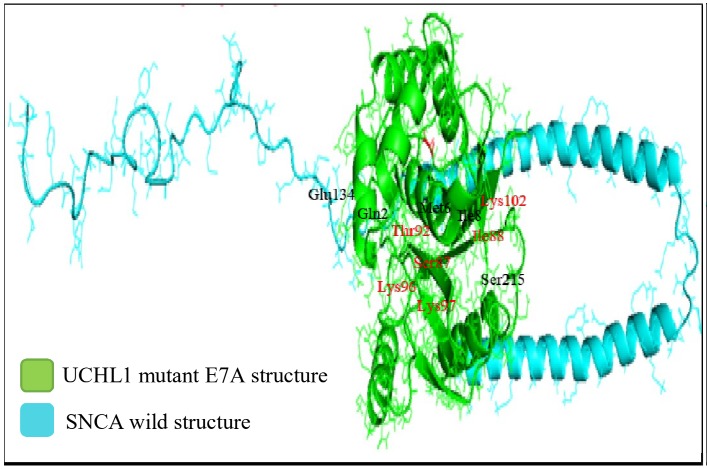

(b) UCHL1 Mutant (S18Y) and SNCA wild type Complex

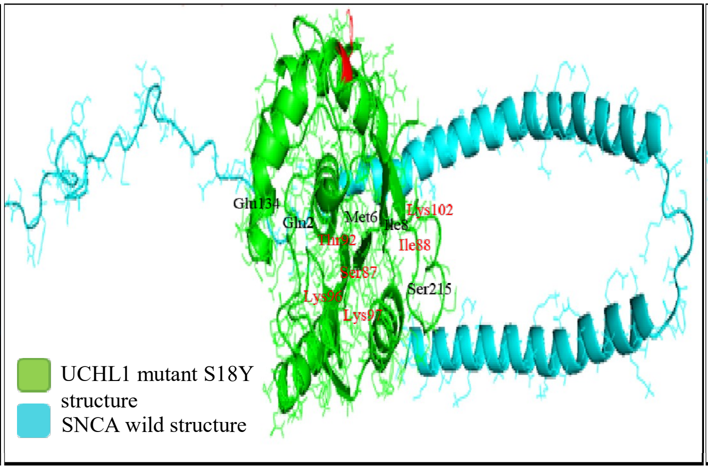

(c) UCHL1 Mutant (I93M) and SNCA wild type Complex

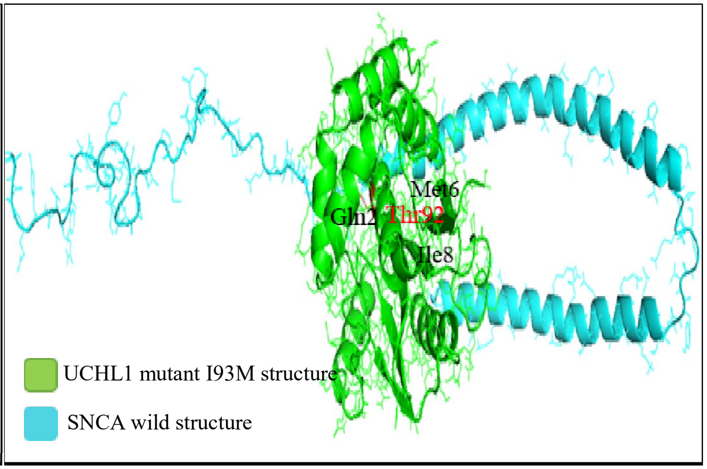

(d) UCHL1 Mutant (R178Q) and SNCA wild type Complex

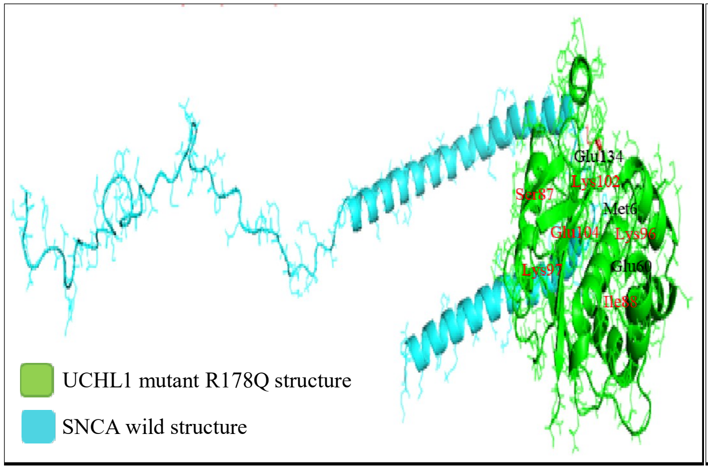

(e) UCHL1 Mutant (A216D) and SNCA wild type Complex

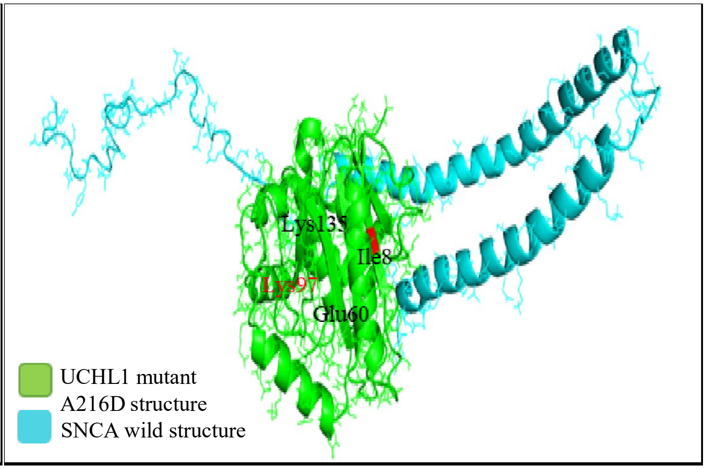

Figure S4 Interactions analysis of wild-type SNCA protein with five PD and other neurological disorders causing mutant versions of UCHL1 proteins. (a)Diagram displaying interactions between the human wild type SNCA protein with PD causing mutant version of UCHL1 (E7A).(b) It represents interaction analysis of wild-type SNCA with PD causing mutant version of UCHL1(S18Y). (c) It represents interaction analysis of wild-type SNCA with PD causing mutant version of UCHL1(I93M). (d) It revels interaction analysis of wild-type SNCA with PD causing mutant version of UCHL1(R178Q).(e) Diagram represents interaction analysis of wild-type SNCA with PD causing mutant version of UCHL1(A216D). In penal (a),(b),(c),(d) and (e) interacting partner are color coded and their interacting residues are highlighted in black color for PD causing mutant versions of UCHL1 and red for wild-type SNCA.

Supplementary Figure S5

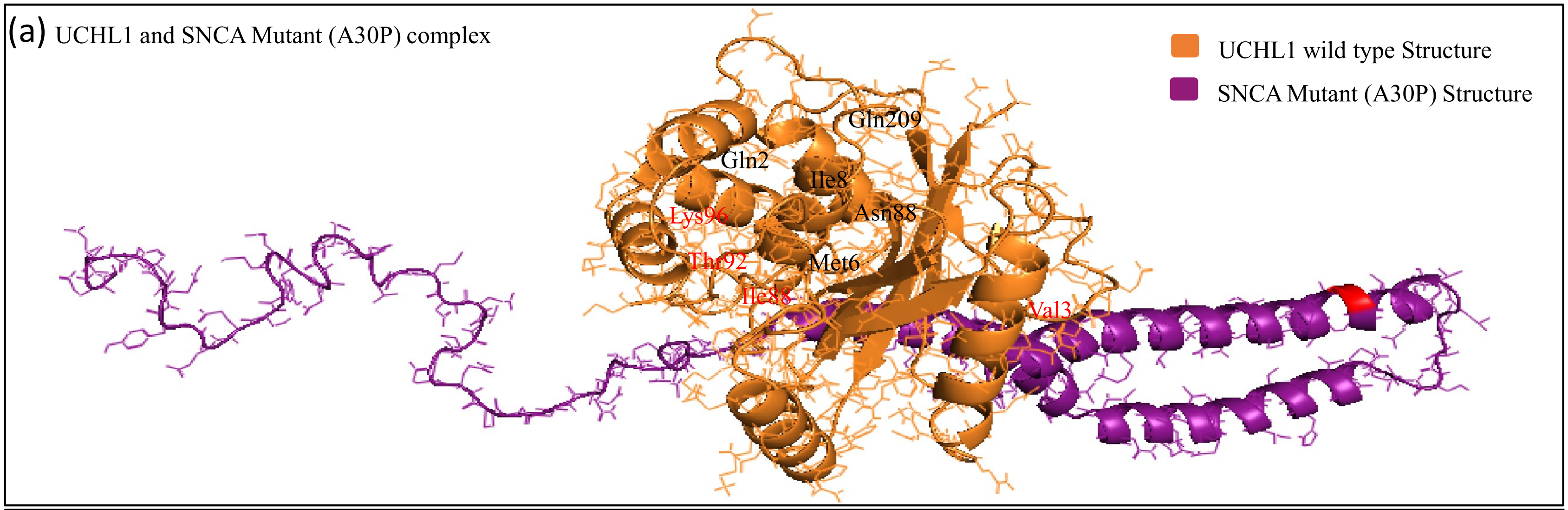

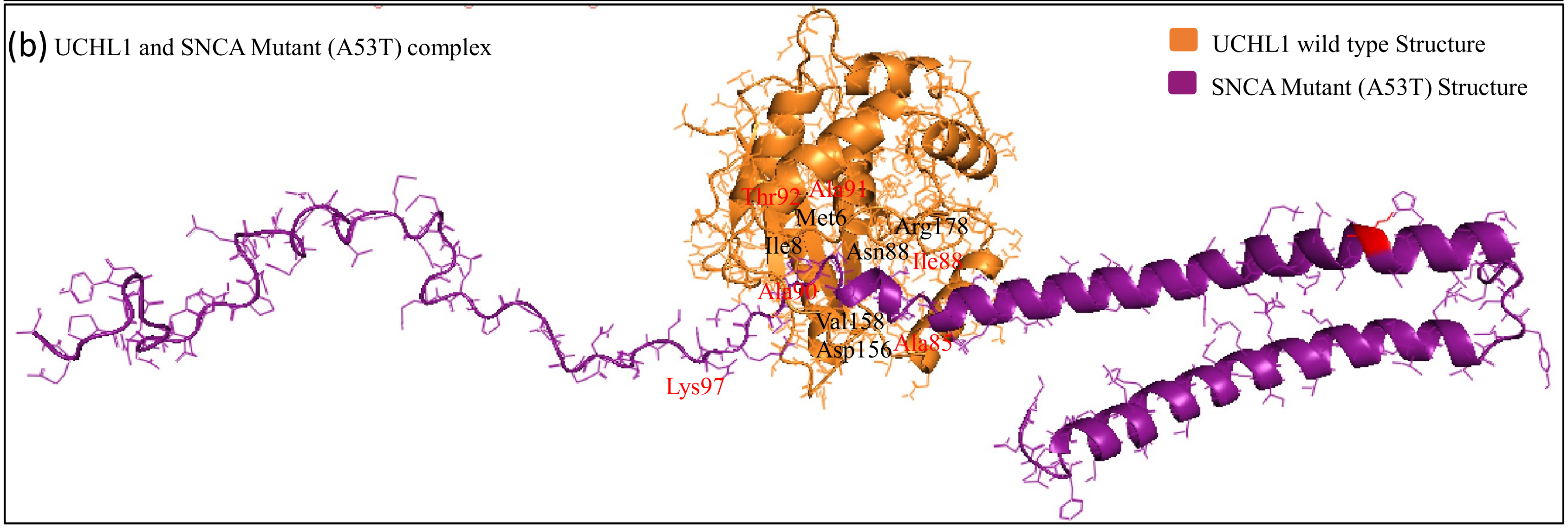

Figure S5. Interactions analysis of wild-type UCHL1 proteins with two reported PD and causing mutant versions of SNCA protein structures.(a) Diagram displaying interactions between the Wild type UCHL1 proteins with mutant version of SNCA (A30P). (b) Diagram displaying interactions between the Wild type UCHL1 proteins with mutant version of SNCA (A53T). In both penal (a) and (b), interacting partners are color coded and interacting residues are highlighted in black for wild type UCHL1 protein and red for mutant SNCA proteins.

Supplementary Figure S6

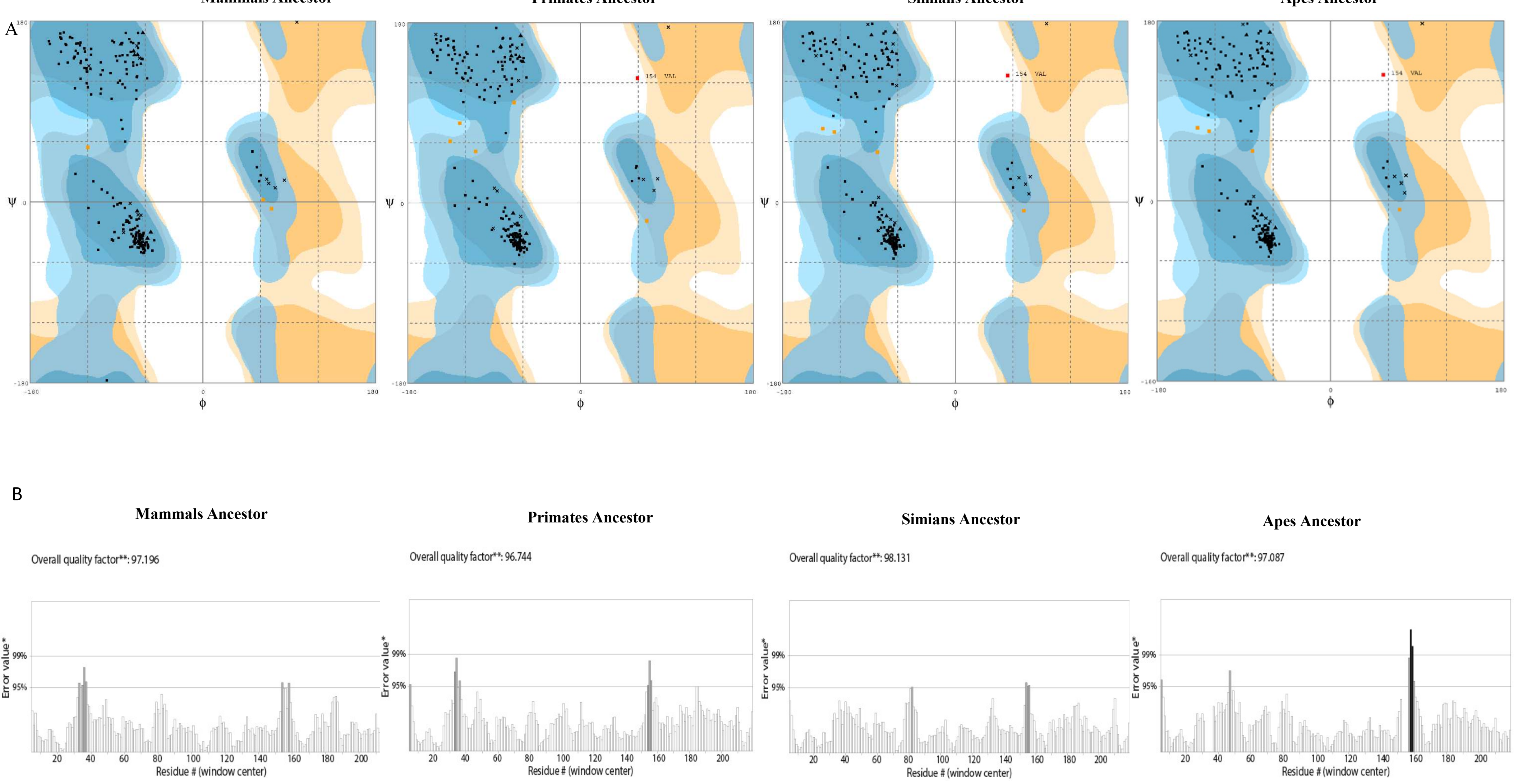

Figure S6 .Evaluation of 3D models of UCHL1 proteins. (A) Ramachandran plots of mammalian ancestral, primates ancestral, simians ancestral and apes ancestral specific UCHL1 proteins. (B) Quality of models have been examined with the help of Errat. Overall quality factor is expressed as percentage of the protein for which the calculated error value falls below the 95% rejection limit, calculated by Errat.

Supplementary Figure S7

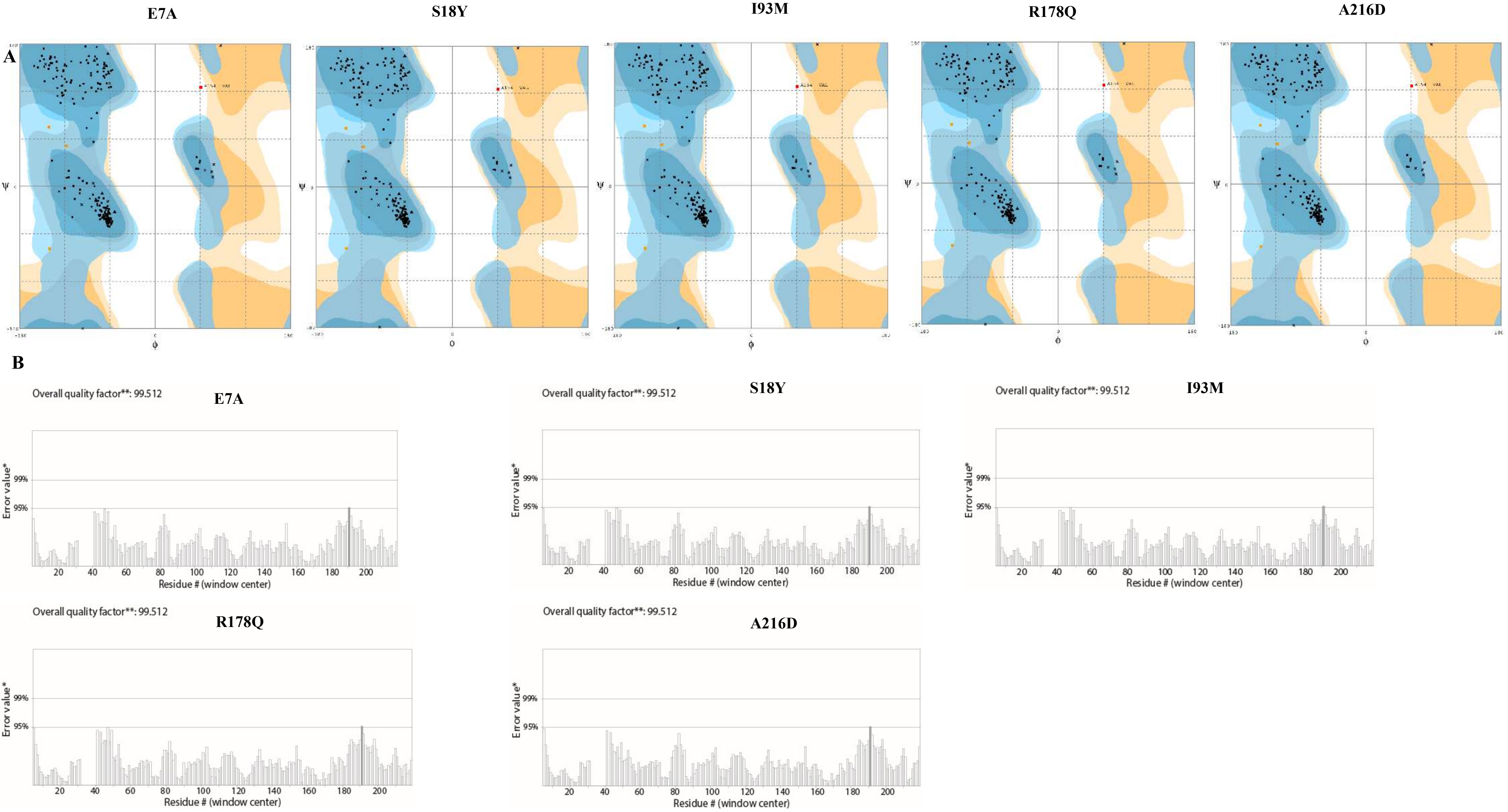

Figure S7. **Evaluation of 3D models of mutant UCHL1 proteins.** (A) Ramachandran plots of human specific disease causing missence mutation of UCHL1 involved in PD and other neurological disorders. (B) Quality of models have been examined with the help of Errat. Overall quality factor is expressed as percentage of the protein for which the calculated error value falls below the 95% rejection limit, calculated by Errat.
