# Supplementary material for: Molecular evolutionary and structural analysis of human UCHL1 gene demonstrates the relevant role of intragenic epistasis in Parkinson’s disease and other neurological disorders
Source: BMC Evol Biol. 2020 Oct 7;20:130. doi: 10.1186/s12862-020-01684-7 (PMC7542113; doi:10.1186/s12862-020-01684-7)
Supplement: Supplementary file 2 — Additional file 2. Supplementary Tables. [file 12862_2020_1684_MOESM2_ESM.docx]

**Additional file 2 for: Molecular evolutionary and structural analysis of human *UCHL1* gene demonstrates the relevant role of intragenic epistasis in Parkinson’s disease and other neurological disorders**

# Muhammad Saqib Nawaz^1^, Razia Asghar^1^ , Nashaiman Pervaiz^1^,Shahid Ali^1^, Irfan Hussain^1^, Peiqi Xing^2^,Yiming Bao^2^* & Amir Ali Abbasi^1^*

1National Center for Bioinformatics, Program of Comparative and Evolutionary Genomics, Faculty of Biological Sciences, Quaid-i-Azam University, Islamabad 45320, Pakistan

2National Genomics Data Center; BIG Data Center & CAS Key Laboratory of Genome Sciences and Information, Beijing Institute of Genomics, Chinese Academy of Sciences, Beijing, 100101, China

*Corresponding authors

Y.B*:E-mail: baoym@big.ac.cn

A.A.A*:E-mail:[abbasiam@qau.edu.pk](mailto:abbasiam@qau.edu.pk)

**Additional file 2: Supplementary Tables.**

**Table S1. Estimation of number of synonymous substitutions per synonymous site (dS), number of non-synonymous substitutions per non-synonymous site (dN) with Hyphy.**

| **Substitution Model/Method: Codon Based (GY-94)** | | |
| --- | --- | --- |
| **Species-UCHL1** | **dN/dS(dN,dS) dN-dS1** | |
| **Hominoids** |  | |
| Human | 0(0,0.0069) | -0.0487 |
| Chimpanzee | 0(0,0.0069) |  |
| Gorilla | 0(0,0.0069) |  |
| Orangutan | 0(0,0.028) |  |
| **Non-Hominoids** |  | |
| Macaque | 0.0443(0.0059,0.1331) | -2.6191 |
| Squirrel Monkey | 0(0,0.0133) |  |
| Marmoset | 0(0,0.1068) |  |
| Otolemur | 0.0157(0.0379,2.4097) |  |
| **Non-Primate Placental Mammals** |  | |
| Cow | 0.0368(0.0187,0.5077) | -3.0412 |
| Cat | 0.0350(0.0124,0.3544) |  |
| Elephant | 0.1081(0.0392,0.3627) |  |
| Mouse | 0.0143(0.0274,1.9141) |  |
| **Non-Mammalian Tetrapods** |  | |
| Chicken | 0.1080 (0.0225,0.2083) | -6.3294 |
| Zebra Finch | 0.0516 (0.0337,0.6527) |  |
| Turtle | 0.1249(0.0745,0.5967) |  |
| Coelacanth | 0.0831 (0.4532,5.4556) |  |
| ^1^dN-dS<0 implies negative selection constraint on UCHL1 within sarcopterygian lineage. | | |

**Table S2. Identification of negatively constrained sites in UCHL1 among sarcopterygians at 0.1 significance level with Hyphy.**

| **Index** | **Residue Number** | **dN-dS** | **p-value** |
| --- | --- | --- | --- |
| 1 | 4 | -10.227823 | 0.000448 |
| 2 | 5 | -4.000000 | 0.012346 |
| 3 | 8 | -6.088260 | 0.001537 |
| 4 | 10 | -3.000000 | 0.037037 |
| 5 | 19 | -4.434942 | 0.059223 |
| 6 | 22 | -4.021801 | 0.044513 |
| 7 | 24 | -4.495744 | 0.042811 |
| 8 | 28 | -5.322538 | 0.027984 |
| 9 | 30 | -4.078550 | 0.057874 |
| 10 | 31 | -3.993461 | 0.030172 |
| 11 | 36 | -4.459431 | 0.058209 |
| 12 | 41 | -4.642451 | 0.075728 |
| 13 | 43 | -4.814949 | 0.022832 |
| 14 | 45 | -6.500000 | 0.002591 |
| 15 | 47 | -4.199331 | 0.048428 |
| 16 | 48 | -6.390299 | 0.006531 |
| 17 | 51 | -2.475238 | 0.084277 |
| 18 | 53 | -2.729332 | 0.093362 |
| 19 | 54 | -5.433681 | 0.013981 |
| 20 | 55 | -3.472562 | 0.046345 |
| 21 | 56 | -3.894333 | 0.060372 |
| 22 | 57 | -4.339156 | 0.050978 |
| 23 | 60 | -4.168716 | 0.066223 |
| 24 | 65 | -6.123580 | 0.018240 |
| 25 | 66 | -4.845409 | 0.037412 |
| 26 | 68 | -4.274670 | 0.079580 |
| 27 | 69 | -10.181914 | 0.000393 |
| 28 | 76 | -5.116095 | 0.031097 |
| 29 | 77 | -3.861246 | 0.069526 |
| 30 | 78 | -5.345838 | 0.037190 |
| 31 | 79 | -5.472506 | 0.016786 |
| 32 | 80 | -4.916572 | 0.050097 |
| 33 | 81 | -4.291630 | 0.035066 |
| 34 | 85 | -3.867172 | 0.080584 |
| 35 | 86 | -4.591539 | 0.052665 |
| 36 | 87 | -5.240485 | 0.042442 |
| 37 | 91 | -8.356780 | 0.000511 |
| 38 | 92 | -7.285972 | 0.002682 |
| 39 | 93 | -4.285539 | 0.052297 |
| 40 | 94 | -6.017217 | 0.015416 |
| 41 | 95 | -5.799628 | 0.009818 |
| 42 | 96 | -7.187661 | 0.004020 |
| 43 | 97 | -7.484344 | 0.003320 |
| 44 | 99 | -3.016897 | 0.098910 |
| 45 | 101 | -4.372276 | 0.039334 |
| 46 | 102 | -7.725347 | 0.000467 |
| 47 | 104 | -5.554325 | 0.022635 |
| 48 | 121 | -5.864676 | 0.010945 |
| 49 | 125 | -4.741053 | 0.026009 |
| 50 | 126 | -5.000000 | 0.019662 |
| 51 | 131 | -3.432736 | 0.093853 |
| 52 | 136 | -7.580294 | 0.002102 |
| 53 | 139 | -4.812001 | 0.024774 |
| 54 | 144 | -3.599718 | 0.078249 |
| 55 | 145 | -6.308376 | 0.010904 |
| 56 | 147 | -8.500000 | 0.000356 |
| 57 | 151 | -7.008421 | 0.020970 |
| 58 | 156 | -3.845648 | 0.068809 |
| 59 | 162 | -5.286529 | 0.040790 |
| 60 | 163 | -3.171718 | 0.099877 |
| 61 | 164 | -4.126057 | 0.058506 |
| 62 | 166 | -4.231468 | 0.083236 |
| 63 | 167 | -4.303052 | 0.059578 |
| 64 | 171 | -5.725529 | 0.036591 |
| 65 | 172 | -4.179141 | 0.073594 |
| 66 | 179 | -5.155830 | 0.052343 |
| 67 | 181 | -5.319483 | 0.044877 |
| 68 | 182 | -4.284569 | 0.073808 |
| 69 | 185 | -5.222212 | 0.023683 |
| 70 | 186 | -5.396397 | 0.032087 |
| 71 | 194 | -3.362055 | 0.032355 |
| 72 | 197 | -4.236068 | 0.053998 |
| 73 | 205 | -5.357954 | 0.032840 |
| 74 | 209 | -4.345125 | 0.070403 |
| 75 | 211 | -4.152873 | 0.051715 |
| 76 | 212 | -6.232802 | 0.014620 |
| 77 | 216 | -6.994824 | 0.003489 |
| 78 | 218 | -6.000000 | 0.008281 |
| Abbreviations: dS, synonymous substitutions per synonymous site; dN, non-synonymous substitutions per non-synonymous site. 4th column depicts p-value (p<0.1) suggesting putative negatively constrained sites. | | | |

**Table S3. Structural deviation analysis in the back bone torsion angles of the five disease causing mutant versions of UCHL1 proteins; incorporating PD and other neurological disease causing mutations (employing I‐TASSER server).**

| **Mutations** | **Major Changes in Residue Number** | **Major shifts in region Critical Region** |
| --- | --- | --- |
| Glu7Ala | 1-2,5-8,23-25 | C12-peptidase Domain |
|  | 32-47 | Secretion signal Motif |
|  | 85-89,102-108,123-125 | 32-39 (Secretion |
|  | 147-155,177-179,187, 191-192, | Signal Motif) |
|  | 222-223 | Farnesylation site |
|  |  |  |
| Ser18Tyr | 1-8, | C12- peptidase Domain |
|  | 32-46 | Secretion signal Motif 32-39(Secretion |
|  | 55,85-89,102-108,123-124,134-137.148-156, | Signal Motif) |
|  | 176-179,208-215 |  |
|  | 221-223 | Farnesylation site |
|  |  |  |
| Ile93Met | 1-2,5-6,11-20,  36-47, | C12 peptidase Domain  Secretion signal Motif |
|  | 86-90,100-108,110-112, |  |
|  | 136,147-150,154-156,179-180,184-185,209-212 | 32-39(Secretion  Signal Motif) |
|  | 222-223 | Farnesylation site |
|  |  |  |
| Arg178Gln | 5-10, | C12-peptidase Domain |
|  | 33-47 | Secretion signal Motif 32-39(Secretion |
|  | 123-124,144-158,178-180,183-187, 209-215, | Signal Motif) |
|  | 222-223 | Farnesylation site |
|  |  |  |
| Ala216Asp | 1-2,5-6,23-25, | C12-peptidase Domain |
|  | 30-49, 54-56,68-73,85-90, | Secretion signal Motif |
|  | 110-111, 123-124,135,152-156,179-180,209-215 | 32-39(Secretion  Signal Motif) |
|  | 222-223 | Farnesylation site |
| This table shows the impact of missense mutations in UCHL1 protein on its backbone torsion angles by comparing them with its wild-type protein structure. In the first column, amino acid residue on the left indicates the wild-type residue; the number shows the amino acid position of the residue in the protein sequence, while the residue on the right shows the mutated residue. The second column specifies the positions at which major structural deviations were observed. The third column depicts the deviated region/residues shared among all mutant proteins analyzed (critical region). | | |

**Table S4. Structural deviation analysis in the back bone torsion angles of the five disease causing mutant versions of UCHL1; incorporating PD and other neurological disease causing mutations (using the Robetta server).**

| **Mutations** | **Major Changes in Residue Number** | **Major shifts in region Critical Region** |
| --- | --- | --- |
| Glu7Ala | 4,7, | C12-peptidase Domain |
|  | 31-35 | Secretion signal Motif |
|  | 45,102-103,121-124 | 32-39 (Secretion |
|  | 148-150,181,190-192, | Signal Motif) |
|  | 221-223 | Farnesylation site |
|  |  |  |
| Ser18Tyr | 6-7, 19-21, | C12- peptidase Domain |
|  | 31-39 | Secretion signal Motif 32-39(Secretion |
|  | 56-63,73,88-89,104-106,120-121,149-153,157-160, | Signal Motif) |
|  | 178,183-191,203-212 |  |
|  |  |  |
| Ile93Met | 5,25,  31-39, | C12 peptidase Domain  Secretion signal Motif |
|  | 45,90-92,110-111,123-124, |  |
|  | 136,148-150,155-158,206-210, 213-214, | 32-39(Secretion  Signal Motif) |
|  | 221-223 | Farnesylation site |
|  |  |  |
| Arg178Gln |  | C12-peptidase Domain |
|  | 30-41 | Secretion signal Motif 32-39(Secretion |
|  | 88-90,101-105, 110-112,121-124,191-192, 207-211, | Signal Motif) |
|  | 221-223 | Farnesylation site |
|  |  |  |
| Ala216Asp | 25, | C12-peptidase Domain |
|  | 30-33, | Secretion signal Motif |
|  | 88-91,110-111,149-151,166-172,189-193,206-210 | 32-39(Secretion  Signal Motif) |
|  | 221-223 | Farnesylation site |
| This table shows the impact of missense mutations in UCHL1 protein on its backbone torsion angles by comparing them with its wild-type protein structure. In the first column, amino acid residue on the left indicates the wild-type residue; the number shows the amino acid position of the residue in the protein sequence, while the residue on the right shows the mutated residue. The second column specifies the positions at which major structural deviations were observed. The third column depicts the deviated region/residues shared among all mutant proteins analyzed (critical region). | | |

**Table S5. Analysis of interacting residues involved in hydrogen bonding between the docked complexes of UCHL1, SNCA and PARKN.**

| **Docked Complex** | **Binding energy**  **(kcal/mole)** | **Interacting residues of UCHL1** |  | **Interacting residues of SNCA/PARKIN** | **Hydrogen Bonding(Å)** |
| --- | --- | --- | --- | --- | --- |
| **Human UCHL1-SNCA complex** | -864.11 | Gln2 |  | Lys96 | 2.65 |
|  |  | Met6 |  | Thr92 | 2.63 |
|  |  | Asn88 |  | Thr92 | 2.71 |
|  |  | Ile8 |  | Ile88 | 2.74 |
|  |  | Gln209 |  | Val3 | 3.17 |
| **Human UCHL1-PARKIN complex** | -743.7 | Glu7 |  | Gly335 | 3.01 |
|  |  | Ile8 |  | Gly359 | 2.87 |
|  |  | Asp30 |  | Gly361 | 3.0 |
|  |  | Leu32 |  | Arg334 | 2.75 |
|  |  | Gln209 |  | Glu310 | 2.93 |
|  |  | Arg213 |  | Gln317 | 2.65 |
|  |  | Phe214 |  | Cys337 | 2.92 |
|  |  | Ser215 |  | Leu358 | 3.19 |
|  |  | Ser215 |  | Gly359 | 2.67 |
|  |  | Cys152 |  | Leu342 | 3.30 |
|  |  | Gln151 |  | Glu344 | 2.84 |
|  |  | Gln151 |  | Gln345 | 3.08 |
|  |  | Arg153 |  | Asn356 | 2.97 |
|  |  | Val154 |  | Asn356 | 2.74 |
| This table depicts interaction on the basis of hydrogen bonding between UCHL1 protein with SNCA and PARKIN. Second column depicts the lowest binding energies of the docked complexes on kcal/mol. Fifth column represents hydrogen bonding between the hydrogen bond donor and hydrogen bond acceptor in Angstrom in docked complexes. | | | | | |

**Table S6. Analysis of interacting residues involved in hydrogen bonding between the docked complexes of UCHL1 and SNCA.**

| **Docked Complex** | **Binding energy**  **(kcal/mole)** | **Interacting residues of UCHL1** | **Interacting residues of SNCA** | **Hydrogen Bonding(Å)** |
| --- | --- | --- | --- | --- |
| **Human UCHL1(I93M mutant)-SNCA complex** | -778.8 | Gln2 | Lys96 | 2.64 |
|  |  | Met6 | Thr92 | 2.64 |
|  |  | Ile8 | Ile88 | 2.80 |
|  |  | Ser215 | Ser87 | 3.28 |
|  |  | Lys135 | Glu104 | 2.64 |
|  |  | Gln209 | Val3 | 2.82 |
|  |  | Glu60 | Lys97 | 2.53 |
| **HumanUCHL1**  **(E7A mutant)-**  **SNCA** | -791.9 | Lys135 | Glu104 | 2.73 |
|  |  | Met6 | Thr92 | 2.65 |
|  |  | Ser215 | Ser87 | 3.01 |
|  |  | ILe8 | Ile88 | 2.75 |
|  |  | Gln2 | Lys96 | 2.65 |
|  |  | Gln209 | Val3 | 2.86 |
|  |  | Glu60 | Lys97 | 2.72 |
|  |  | Glu134 | Lys102 | 2.72 |
| **HumanUCHL1**  **(S18Y) - SNCA** | -763.6 | Gln209 | Val3 | 2.86 |
|  |  | Gln2 | Lys96 | 2.65 |
|  |  | ILe8 | Ile88 | 2.80 |
|  |  | Lys135 | Glu104 | 2.65 |
|  |  | Met6 | Thr92 | 2.64 |
|  |  | Glu60 | Lys97 | 2.49 |
|  |  | Glu7 | Lys34 | 2.49 |
| **Human UCHL1**  **(R178Q)**-  **SNCA** | -916.8 | Ser215 | Thr33 | 2.70 |
|  |  | Glu37 | Thr22 | 2.66 |
|  |  | Ser38 | Thr22 | 2.79 |
|  |  | Val212 | Lys32 | 3.09 |
|  |  | Arg213 | Lys43 | 2.61 |
|  |  | Arg213 | Val40 | 2.64 |
|  |  | Phe214 | Lys32 | 2.71 |
|  |  | Glu208 | Lys45 | 2.58 |
|  |  | Glu211 | Lys45 | 2.71 |
| **HumanUCHL1**  **(A216D)- SNCA** | -779.7 | Glu7 | Phe4 | 3.13 |
|  |  | Lys15 | Glu83 | 2.70 |
|  |  | Arg19 | Val195 | 3.08 |
|  |  | Glu109 | Thr92 | 2.99 |
|  |  | Glu137 | Gln99 | 2.95 |
| **Human UCHL1**  **-SNCA Mutant (A30P)** | -734.6 | Gln2 | Lys96 | 2.65 |
|  |  | Met6 | Thr92 | 2.63 |
|  |  | Ile8 | Ile88 | 2.74 |
|  |  | Asn88 | Thr92 | 2.71 |
|  |  | Gln209 | Val3 | 3.17 |
| **Human**  **UCHL1-SNCA**  **Mutant (A53T)** | -724.8 | Met6 | Ala91 | 2.85 |
|  |  | Ile8 | Thr92 | 3.04 |
|  |  | Asn88 | Ile88 | 3.00 |
|  |  | Asp156 | Lys97 | 2.54 |
|  |  | Val158 | Ala90 | 2.74 |
|  |  | Arg178 | Ala85 | 2.79 |
| This table depicts interaction on the basis of hydrogen bonding between UCHL1 mutant proteins with SNCA and SNCA mutants (A30P and A53T) interaction with UCHL1 wild type protein. Second column depicts the lowest binding energies of the docked complexes on kcal/mol. Fifth column represents hydrogen bonding between the hydrogen bond donor and hydrogen bond acceptor in Angstrom in docked complexes. | | | | |
