# Supplementary material for: Molecular evolutionary and structural analysis of human UCHL1 gene demonstrates the relevant role of intragenic epistasis in Parkinson’s disease and other neurological disorders
Source: BMC Evol Biol. 2020 Oct 7;20:130. doi: 10.1186/s12862-020-01684-7 (PMC7542113; doi:10.1186/s12862-020-01684-7)
Supplement: Supplementary file 3 — Additional file 3. Complete list of protein sequences used in this study. [file 12862_2020_1684_MOESM3_ESM.pdf]

Additional file 3 for: Molecular evolutionary and structural analysis of human *UCHL1* gene demonstrates the relevant role of intragenic epistasis in Parkinson's disease and other neurological disorders

Muhammad Saqib Nawaz[1], Razia Asghar[1] , Nashaiman Pervaiz[1],Shahid Ali[1], Irfan Hussain[1], Peiqi Xing[2],Yiming Bao[2]* & Amir Ali Abbasi[1]*

1National Center for Bioinformatics, Program of Comparative and Evolutionary Genomics, Faculty of Biological Sciences, Quaid-i-Azam University, Islamabad 45320, Pakistan

2National Genomics Data Center; BIG Data Center & CAS Key Laboratory of Genome Sciences and Information, Beijing Institute of Genomics, Chinese Academy of Sciences, Beijing, 100101, China

*Corresponding authors

Y.B*:E-mail: baoym@big.ac.cn

A.A.A*:E-mail:abbasiam@qau.edu.pk

Additional file 3: Complete list of protein sequences used in this study.

UCHL1

>Homo sapiens Uchl1
MQLKPMEINPEMLNKVLSRLGVAGQWRFVDVLGLEEESLGSVPAPACALLLLFPLTAQHENFRKKQIEELKGQEVSPKV
YFMKQTIGNSCGTIGLIHAVANNQDKLGFEDGSVLKQFLSETEKMSPEDRAKCFEKNEAIQAAHDAVAQEGQCRVDDK
VNFHFILFNNVDGHLYELDGRMPFPVNHGASSEDTLLKDAAKVCREFTEREQGEVRFSAVALCKAA
>Pan troglodytes Uchl1
MQLKPMEINPEMLNKVLSRLGVAGQWRFVDVLGLEEESLGSVPAPACALLLLFPLTAQHENFRKKQIEELKGQEVSPKV
YFMKQTIGNSCGTIGLIHAVANNQDKLGFEDGSVLKQFLSETEKMSPEDRAKCFEKNEAIQAAHDAVAQEGQCRVDDK
VNFHFILFNNVDGHLYELDGRMPFPVNHGASSEDTLLKDAAKVCREFTEREQGEVRFSAVALCKAA
>Gorilla gorilla Uchl1
MQLKPMEINPEMLNKVLSRLGVAGQWRFVDVLGLEEESLGSVPAPACALLLLFPLTAQHENFRKKQIEELKGQEVSPKV
YFMKQTIGNSCGTIGLIHAVANNQDKLGFEDGSVLKQFLSETEKMSPEDRAKCFEKNEAIQAAHDAVAQEGQCRVDDK
VNFHFILFNNVDGHLYELDGRMPFPVNHGASSEDTLLKDAAKVCREFTEREQGEVRFSAVALCKAA
>Macaca mulatta Uchl1
MQLKPMEINPEMLNKVLSRLGVAGQWRFVDVLGLEEESLGSVPAPACALLLLFPLTAQHENFRKKQIEELKGQEVSPKV
YFMKQTIGNSCGTIGLIHAVANNQDKLGFEDGSVLKQFLSETEKMSPEDRAKCFEKNEAIQAAHDAVAQEGQCRVDDK
VNFHFILFNNVDGHLYELDGRMPFPVNHGASSEGTLLQDAAKVCREFTEREQGEVRFSAVALCKAA
>Cebus capucinus Uchl1
MQLKPMEINPEMLNKVLSRLGVAGQWRFVDVLGLEEESLGSVPAPACALLLLFPLTAQHENFRKKQIEELKGQEVSPKV
YFMKQTIGNSCGTIGLIHAVANNQDKLGFEDGSVLKQFLSETEKMSPEDRAKCFEKNEAIQAAHDAVAQEGQCRVDDK
VNFHFILFNNVDGHLYELDGRMPFPVNHGTSSEDTLLQDAAKVCREFTEREQGEVRFSAVALCKAA
>Otolemur garnettii Uchl1
MQLKPMEINPEMLNKVLARLGVAGQWRFADVLGLEEESLGSVPAPACALLLLFPLTAQHENFRKKQIEELKGQEVSPV
YFMKQTIGNSCGTIGLIHAVANNRDKLEFEDGSVLKQFLSETEKMSPEDRAKCFEKNEAIQAAHDAVAQEGQCRVDDK
VNFHFILFNNVDGHLYELDGRMPFPVNHGASSEDCLLQDAAKVCREFTEREQGEVRFSAVALCKAA
>Mus musculus Uchl1
MQLKPMEINPEMLNKVLAKLGVAGQWRFADVLGLEEETLGSVPSPACALLLLFPLTAQHENFRKKQIEELKGQEVSPKV
YFMKQTIGNSCGTIGLIHAVANNQDKLEFEDGSVLKQFLSETEKLSPEDRAKCFEKNEAIQAAHDSVAQEGQCRVDDKV
NFHFILFNNVDGHLYELDGRMPFPVNHGASSEDSLLQDAAKVCREFTEREQGEVRFSAVALCKAA
>Rattus norvegicus Uchl1
MQLKPMEINPEMLNKVLAKLGVAGQWRFADVLGLEEETLGSVPSPACALLLLFPLTAQHENFRKKQIEELKGQEVSPKV
YFMKQTIGNSCGTIGLIHAVANNQDKLEFEDGSVLKQFLSETEKLSPEDRAKCFEKNEAIQAAHDSVAQEGQCRVDDKV
NFHFILFNNVDGHLYELDGRMPFPVNHGASSEDSLLQDAAKVCREFTEREQGEVRFSAVALCKAA
>Equus caballus Uchl1
MQLKPMEINPEMLNKVLARLGVAGQWRFVDVLGLEEETLGSVPAPACALLLLFPLTAQHENFRKKQIEELKGQEVSPK
VYFMKQTIGNSCGTIGLIHAVANNQDKLEFEDGSVLKQFLSETEKLSPEDRAKCFEKNEAIQAAHDAVAQEGQCRVDDK
VNFHFILFNNVDGHLYELDGRMPFPVNHGASSEDLLLQDAAKVCREFTEREQGEVRFSAVALCKAA
>Tursiops truncatus Uchl1
MQLKPMEINPEMLNKVLARLGVAGQWRFEDVLGLEEESLGSVPAPACALLLLFPLTAQHENFRKKQIEELKGQEVSPKV
YFMKQTIGNSCGTIGLIHAVANNQDKLEFEDGSVLKQFLSETEKLSPEDRAKCFEKNEAIQAAHDAVAQEGQCRVDDKV
NFHFILFNNVDGHLYELDGRMPFPVNHGASSEGSLLQDAAKVCREFTEREQGEVRFSAVALCKAA
>Bos taurus Uchl1

MQLKPMEINPEMLNKVLTRLGVAGQWRFEDVLGLEEESLGSVPAPACALLLLFPLTAQHENFRKKQIEELKGQEVSPKV
YFMKQTIGNSCGTIGLIHAVANNQDKLEFEDGSVLKQFLSETEKLSPEDRAKCFEKNEAIQAAHDAVAQEGQCRVDDKV
NFHFILFNNVDGHLYELDGRMPFPVNHGTSSEDSLLQDAAKVCREFTEREQGEVRFSAVALCKAA
>Felis catus Uchl1
MQLKPMEINPEMLNKVLSRLGVAGQWRFADVLGLEEEALGSVPAPACALLLLFPLTAQHENFRKKQIEELKGQEVSPK
VYFMKQTIGNSCGTIGLIHAVANNQDKLEFEDGSVLKQFLSETEKLSPEDRAKCFEKNEAIQAAHDAVAQEGQCRVDDK
VNFHFILFNNVDGHLYELDGRMPFPVNHGTSSEDSLLQDAAKVCREFTEREQGEVRFSAVALCKAA
>Canis lupus familiaris Uchl1
MQLKPMEINPEMLNKQVLARLGVAGQWRFADVLGLEDEALGSVPAPACALLLLFPLTAQHENFRKKQIEELKGQEVSP
KVYFMKQTIGNSCGTIGLIHAVANNQDKLEFEDGSVLKQFLSETEKLSPEDRAKCFEKNEAIQAAHDAVAQEGQCRVDD
KVNFHFILFNNVDGHLYELDGRMPFPVNHGTSSEDSLLQDAAKVCREFTEREQGEVRFSAVALCKAA
>Pteropus vampyrus Uchl1
MQLKPMEINPEMLNKVLARLGVAGQWRFVDVLGLEDEALSSVPAPACALLLLFPLTAQHENFRKKQIEELKGQEVSPK
VYFMKQTIGNSCGTIGLIHAVANNQDKLEFDDGSVLRQFLSETEKLSPEDRAKCFEKNEAIQAAHDAVAQEGQCRVDD
KVNFHFILFNNVDGHLYELDGRMPFPVNHGSSSEDLLLQDAAKVCREFTEREQGEVRFSAVALCKAA
>Myotis lucifugus Uchl1
MQLKPMEINPEMLNKVLARLGVAGQWRFVDVLGLEEDALSSVPAPVCALLLLFPLTAQHENFRKKQIEELKGQEVSPK
VYFMKQTIGNSCGTIGLIHAVANNQDKLEFEDGSVLKQFLSETEKLSPEDRAKCFEKNEAIQTAHDAVAQEGQCRVDDK
VNFHFILFNNVDGHLYELDGRMPFPVNHGTSAEDSLLQDAAKVCREFTEREQGEVRFSAVALCKAT
>Erinaceus uropaeus Uchl1
MQLKPMEINPEMLNKXXXXXXXXGQWRFVDVLGLEDESLGSVPAPACALLLLFPLTAQHENFRKKQIEELKGQEVSPK
VYFMKQTIGNSCGTIGLIHAVANNQDKLEFEDGSVLKQFLSETEKLSPEDRAKCFEKNEAIQAAHDAVAQEGQCRVDDK
VNFHFILFNNVDGHLYELDGRMPFPVNHGSSSEDSLMQDAAKVCREFTEREQGEVRFSAVALCKAA
>Echinops telfairi Uchl1
MQLKPMEINPEMLNKVSGVSRRPPGAPGTPRRRRPPGGAPPCAFPRPPEFGWGLGSVPAPACALLLLFPLTAQHENF
RKKQIEELKGQEVSPKVYFMKQTIGNSCGTIGLIHAVANNQDKLAFENGSVLKQFLSETEKMSPEDRAKCFEKNEAIQAA
HDAVAQEGQCRVDDKVNFHFILFNNVEGHLYELDGRMPFPVNHGSSSEDSLLQDAAKVCREFTEREQGEVRFSAVAL
CKAA
>Monodelphis demestica Uchl1
MQLKPMEINPEMLNKVLTRLGVGGDWKFVDVLGLEEDVLGTVPAPACALLLLFPLTAQHENFRKKQIEELKGQEVSPK
VYFMKQTVGNSCGTIGLIHAVANNQDKLNFDDGSVLKQFISETAKLSPEDRAKCFEKNEAIQAAHDAVAQEGQCRVDD
EVNFHFILFNNVDGHLYELDGRMPFPINHGSNSDESVLKGAAEICRQFTEREEGEVRFSAVALCKCA
>Gallus gallus Uchl1
MAWQPMEINPEMLNKVLSRLGVSPGWRFVDVLGFEEEALGAVPSPACALLLLFPLTEQHENFRKQQTEKIKDQEISSK
VYFLKQTVSNSCGTIGLIHAVANNKDKVKLDEGSALKKFLDETADLSPEERAKRFANNKAIQEVHNSVAQEGQCRVEDN
SVNFHFILFANVDGHLYELDGRLPFPVNHGTSSDDLLLKDSAKICRQFTEREKGEVRFSAVAFCKSA
>Anolis carolinensis Uchl1
MAWQPMEINPEMLNKVLSRLGIAPGWRFVDVLGFEEDSLNAVPNPACALLLLFPLTAQHENFRKQQVEELKGQEVSS
KVYFLKQTASNSCGTIGLIHAIANNQDKILFDEGSALKEFLNATADLSPDERAKRLENNKAIQDAHNAVAEEGQCRAEDD
KVNFHFILFASVDGQLYELDGRMPFPINHGASSDDTLLKASAKVCRQFTEREQGEVRFSAVALSKSA
>Latimeria chalumnae Uchl1
MQWQPMEINPEMLNKVLARLGVSGSWHLVDVLGLEEESLASVPTPVCALMLLFPLTAQHESFRKKQMEEVAEKQVN
PKVYFMKQTIVNSCGTVGLVHAVANNQDKLKFEDDSALKQFLTETADMSAGDRAKCLEKNKIIQEAHDAIAKEGLCRV
EEGSVNFHFITFANIDGNLYELDGRMSFPVNHGSTTKDTLLQDAAKICRQFTEREQGEVRFSAVALCKVK
>Oryzias latipes Uchl1
MEWTPMEINPEMLNKIMSKLGVGDSWRFVDVLGLEGEQLSAVPKPCCALMLLFPLTQQHESFRAQQADKVDDGSEV
YFLKQKAGNSCGTIALLHAVANNKDKMAFDAGSALKKFLEETANMSPDDRAKHLEQNKAIYDAHNEVAMQGQCRPE
ADKINFHFIAFINAKGNLYECDGKMDGPVNHGETTEESFLADAAKVCRGFTEREKDEVRFSAVALCRS

>Gasterosteus aculeatus Uchl1
MEWTPMEINPEMLNKMMINLGVGESWRFSDVLGFEKDQLSAVPKPCCALMLLFPLTQQHESFRQQQADSVTDGSG
VYFLKQTASNSCGTIAMLHAVANNKSKMSFEGGSALKKFLDETANMSADDRAKHLEKNKAIWEAHNEVAAQGQCRP
EADKVNFHFIAFVNVTGQLYEFDGRMNGPVKHGATKDDSFVMDAAKVCRGFIEREQGEQPLYLCMWSLSDSFIL
>Tetraodon nigroviridis Uchl1
MEWTPMELNPEMLNMLMTSLGVNESWRFVDVVGLESEQLSAVPKPCCALMLLFPLTQQHESFRKQQADKIVEETGV
YFLKQMAPNSCGTVALLHAVANNKGKLTFASDSVLQKFLDETADMSSDDRAKHLEKN
>Takifugu rubripes Uchl1
MEWTAMELNPEMLNLLMKSLGVNESWRFVDVVGLESEQLSAVPKPCCSLMLLFPLTQQHETFRKQQADKIAEDSGV
YFLKQTASNSCGTIALLHAVANNKGKFAFASGSVLEKFLNETANMSPEDRAKHLENNKTIFDAHNEVASQGQCRPAAD
KVNFHFITFVNVNGQLYEFDGRVNGPVKHGSTSDESFIMDAAKVCHGFMEREQGEVRFSSVALCQS
>Danio rerio Uchl1
MEWKPMEINPEMLNKVLSKLGVGSKWRFVDVLGLEDESLSGVPSPCCAMMLLFPLTQQHEDFRSKQSVGDCKDVYF
LKQTVVNSCGTVGLVHAVANNQDSIDFDNNSALKKFLEATSGMSPAERAKELEQNKAIQETHDAVADEGQCRPEADK
VNFHFITFVNVNGRLYELDGRIDGPVSHGPTKPDSFVMDAARVCREFMEREKGEVRFSAVALCKA
>Lepisosteus oculatus Uchl1
STAMEWKPMEINPEMLNKVLSKLGVGDSWRFVDVLGFEDEPLASVPKPSCAVMLLFPLTPQHETFREKQSTELAEKAK
EIPSVYFIKQTIVNSCGTVGLLHAVANNKDKLEFKSGSALKSFLDKTASMSAEDRAKELEKSQAIHTAHDEVAAEGQCRV
EEDKVNFHFITFINVDGHLYELDGRMSVPVDHGATKDDSFIMDAAKICRQFVEREKGEVRFSAVALCKAA
>Callorhinchus milii Uchl1
MEWKAMEINPEMLNKCMTRLGVAASWKFVDVLGLETESLSMVPSPVCALLLLFPLSQQHESFRGTQTSELTGKGIDSK
IYFLRQTISNSCGTVGLVHTIANNQDKFTFVEGSTLKKFLTETADLSAEERAKHLEQNKDIHSAHDATAEEGHCRIHEDGT
NFHFIVFVDVDGHLYELDGRMPFPIDHGETSKDTLLQDSAKICRQFMERDQEEVRFTAVALSKA
>Rhincodon typus Uchl1
MEWKPMEINPEMLNKCLSRLGVASSWKFVDVLGLENESLSMVPSPVCALMLLFPLSQQHESYRERQTSELSGKGIDSKI
YFVKQTISNSCGTXGLINTIANNQDKLDFVEGSVLKKFLNETVDLSAEERAKQLEQKQEICAAHDATAEEGECRVHEDGT
NFHFITFVGVDDHLYELDGRMPFPIDHGETSNDQLLQDSAKICRQFMERDQGEVRFTAVALSKAS
